# Immune-checkpoint proteins, cytokines, and microbiome impact on patients with cervical insufficiency and preterm birth

**DOI:** 10.3389/fimmu.2023.1228647

**Published:** 2023-07-24

**Authors:** Seri Jeong, Won Kyong Cho, Yeonhwa Jo, Soo-Ran Choi, Nuri Lee, Kibum Jeon, Min-Jeong Park, Wonkeun Song, Keun-Young Lee

**Affiliations:** ^1^ Department of Laboratory Medicine, Kangnam Sacred Heart Hospital, Hallym University College of Medicine, Seoul, Republic of Korea; ^2^ College of Biotechnology and Bioengineering, Sungkyunkwan University, Suwon, Republic of Korea; ^3^ Department of Obstetrics and Gynecology, Inha University College of Medicine, Inha University Hospital, Incheon, Republic of Korea; ^4^ Department of Laboratory Medicine, Hangang Sacred Heart Hospital, Hallym University College of Medicine, Seoul, Republic of Korea; ^5^ Division of Maternal-Fetal Medicine, Department of Obstetrics and Gynecology, Kangnam Sacred Heart Hospital, Hallym University College of Medicine, Seoul, Republic of Korea

**Keywords:** cervix, cervical insufficiency, cytokines, immune-checkpoint proteins, inflammation, microbiome, preterm birth

## Abstract

**Background:**

Microenvironmental factors, including microbe-induced inflammation and immune-checkpoint proteins that modulate immune cells have been associated with both cervical insufficiency and preterm delivery. These factors are incompletely understood. This study aimed to explore and compare interactions among microbiome and inflammatory factors, such as cytokines and immune-checkpoint proteins, in patients with cervical insufficiency and preterm birth. In particular, factors related to predicting preterm birth were identified and the performance of the combination of these factors was evaluated.

**Methods:**

A total of 220 swab samples from 110 pregnant women, prospectively recruited at the High-Risk Maternal Neonatal Intensive Care Center, were collected between February 2020 and March 2021. This study included 63 patients with cervical insufficiency receiving cerclage and 47 control participants. Endo- and exocervical swabs and fluids were collected simultaneously. Shotgun metagenomic sequencing for the microbiome and the measurement of 34 immune-checkpoint proteins and inflammatory cytokines were performed.

**Results:**

First, we demonstrated that immune-checkpoint proteins, the key immune-regulatory molecules, could be measured in endocervical and exocervical samples. Secondly, we identified significantly different microenvironments in cervical insufficiency and preterm birth, with precise cervical locations, to provide information about practically useful cervical locations in clinical settings. Finally, the presence of *Moraxella osloensis* (odds ratio = 14.785; P = 0.037) and chemokine CC motif ligand 2 levels higher than 73 pg/mL (odds ratio = 40.049; P = 0.005) in endocervical samples were associated with preterm birth. Combining *M. osloensis* and chemokine CC motif ligand 2 yielded excellent performance for predicting preterm birth (area under the receiver operating characteristic curve = 0.846, 95% confidence interval = 0.733-0.925).

**Conclusion:**

Multiple relationships between microbiomes, immune-checkpoint proteins, and inflammatory cytokines in the cervical microenvironment were identified. We focus on these factors to aid in the comprehensive understanding and therapeutic modulation of local microbial and immunologic compositions for the management of cervical insufficiency and preterm birth.

## 1 Introduction

Preterm birth (before 37 completed weeks of gestation) occurs in approximately 15 million cases annually worldwide (1) and is the leading cause of neonatal death (2). Approximately 1.1 million babies die from complications of prematurity. Cervical insufficiency, defined as painless cervical dilation in the second trimester (3), has traditionally been associated with preterm birth (4). If cervical insufficiency is left untreated, most patients deliver within two to three weeks (5), resulting in miscarriage or extremely early preterm birth. Cervical cerclage has been used to treat cervical insufficiency to reduce perinatal mortality and recurrent preterm delivery (6). In particular, emergency cerclage is indicated when visible cervical dilatation or an unexpected finding of a shortened cervix on routine examination is present (7).

The cervix, located between the microbe-rich vaginal environment and the presumably sterile intrauterine space, provides a mechanical barrier for preventing ascending infection by remaining closed during pregnancy (8). The cervix has both innate and adaptive immune functions, mediated by diverse cell types and molecules (9, 10). Patients with cervical insufficiency are therefore vulnerable to ascending infection caused by the disruption of these barriers (8). Microenvironmental factors have been associated with disease pathogenesis and preterm delivery (11, 12). However, our understanding of the interactions among microbial composition, inflammatory factors, immune-checkpoint proteins, and the modulation of immunity in patients with cervical insufficiency and preterm birth is incomplete. Accordingly, we have studied these factors in patients with cervical insufficiency and preterm conditions, stratified by cervical location (endocervix and exocervix), to identify clinical diagnostic and therapeutic targets, particularly their predictive utility.

## 2 Materials and methods

### 2.1 Study design and sample collection

Subjects were pregnant women prospectively recruited at the High-Risk Maternal Neonatal Intensive Care Center in Kangnam Sacred Heart Hospital. Samples were collected between February 2020 and March 2021. All participants provided written informed consent and the study protocol was approved by the Institutional Review Board of Kangnam Sacred Heart Hospital (HKS 2019-11-024 and 2022-06-010). The inclusion criteria for patients with cervical insufficiency undergoing cerclage were as follows: length < 25 mm or internal os dilation of at least 1 cm, effacement of at least 50%, membranes visible at or beyond the external os, and gestational age between 16 and 24 weeks. The exclusion criteria were less than 18 years of age, multifetal gestations, major fetal malformations, ruptured membranes, vaginal bleeding, clinical chorioamnionitis, or persistent, regular contractions. Outpatients visiting our clinical center for the observation of normal pregnancy or for prophylactic cerclage due to history of previous preterm birth during the study period were included. For normal pregnant women as a control group, only gentle swab sampling without cerclage was performed during routine colposcopic examinations. Endo- and exocervical swabs and fluids were collected simultaneously and stored for analysis. The location of the endocervix is around the internal cervical os and the exocervix is near the vaginal fornix. A uniconcave balloon was used for cerclage (13, 14). After identification of the bulging fetal membranes, the cervix was retracted with two atraumatic forceps, and a sufficiently inflated balloon (**Supplementary Figure S1**) was used to push fetal membranes back into the uterus. Sutures were placed as high as possible using the McDonald technique with a 5-mm polyester tape. After deflating the balloon, a purse-string suture was placed and the instrument was withdrawn from the cervix. A physician working under the Maternal-Fetal Medicine division conducted all procedures using the same technique.

### 2.2 Shotgun metagenomic sequencing for microbiome

The OMNIgene OMR-130 kit (DNA Genotek Inc., Ottawa, Canada) was used to analyze swab samples. DNA extraction was performed using the Maxwell 16 LEV Blood DNA Purification Kit (Promega, Madison, WI, USA) within four weeks of sampling, based on the manufacturer’s instructions. After assessing DNA quality using PCR and electrophoresis, libraries were prepared as per standard Illumina protocols. The TruSeq Nano DNA Library Prep kit was used for library preparation, and the quality of the sample pool was confirmed. Libraries were paired-end sequenced (2 × 150 bp) using the Illumina HiSeqXten platform (Illumina, San Diego, CA, USA). After trimming, reads aligned to the human reference genome (GRCh38) were removed using BBDuk software. Finally, clean reads mostly derived from microorganisms were used for metagenomic analyses using Kraken2 (15).

### 2.3 Immune-checkpoint proteins and inflammatory cytokines

Sterile Dacron swabs (Puritan Medical Products, Guilford, ME) were used to take samples from the endo- or exocervix. Soluble immune-checkpoint proteins were quantified using the MILLIPLEX Human Immuno-Oncology Checkpoint Protein Premixed 17-plex Panel (Merck, Darmstadt, Germany). The measured immune-checkpoint proteins were soluble cluster of differentiation 28 (sCD28), soluble T-cell immunoglobulin and mucin-domain containing-3 (sTIM-3), soluble herpes virus entry mediator (sHVEM), sCD40, lymphocyte activation gene 3 (sLAG-3), soluble Toll-like receptor 2 (sTLR-2), soluble programmed death-ligand 1 (sPD-L1), soluble cytotoxic T-lymphocyte-associated protein 4 (sCTLA-4), sCD80/B7-1, sCD86/B7-1, soluble programmed cell death protein 1 (sPD-1), sPD-L2, and soluble B- and T-lymphocyte attenuator (sICOS). Inflammatory cytokines were assayed using the Human XL Cytokine Luminex Performance Panel Premixed Kit (R&D Systems, Minneapolis, MN). The measured cytokines were chemokine CC motif ligand 2 (CCL2), CCL3, CCL4, C-X-C motif chemokine ligand (CXCL10), granulocyte-macrophage colony-stimulating factor (GM-CSF), interferon (IFN)-α, IFN-γ, interleukin (IL)-1α, IL-1β, IL-4, IL-6, IL-8, IL-10, IL-12, IL-13, IL-17A, and tumor necrosis factor-α (TNF-α). All assays were conducted using Luminex-based multiplex technology according to the manufacturer’s protocols on a Bio-Plex 200 instrument (Bio-Rad, Hercules, CA).

### 2.4 Statistical analyses

Principal component analysis (PCA) was conducted to reduce the number of observed variables to a smaller number of principal components accounting for most of the variance in the observed variables. For pre-processing data, the pcaMethods R package was utilized for row scaling (16). The values were divided by standard deviations using the unit variance scaling method. The utilized default method for calculating principal components was singular value decomposition with imputation. The distribution of continuous variables, including mean, mean standard error, standard deviation, skewness, kurtosis, 1st quartile, median, 3rd quartile, and *P*-values for normality, are presented in **Supplementary Table S1**. The Anderson-Darling test was used for normality. For descriptive statistics for comparisons, the Chi-square test was applied to categorical variables and the Wilcoxon test was utilized for paired groups. The Mann-Whitney and Kruskal-Wallis tests were applied to two unpaired groups and three unmatched groups, respectively. For multiple comparisons, nonparametric multiple comparisons for relative effects were performed using the moonBook and nparcomp packages in R for the correction of *P*-values. Multivariate logistic regression analysis was performed, using the factors having a *P* value less than 0.05 in the univariate analysis as co-variables. A receiver operating characteristic (ROC) analysis was conducted to assess the performance of combined factors found to be significant in multivariate analyses to predict preterm birth. The area under the curve (AUC) of combined markers was classified as follows: acceptable (between 0.7 and 0.8), excellent (between 0.8 and 0.9), and outstanding (over 0.9) (17). Spearman’s rank correlation analyses were performed to assess the associations of the microbiome with clinical significance, immune-checkpoint proteins, and inflammatory cytokines. Statistical analyses were performed using ClustVis (a web tool for visualizing clustering of multivariate data) (18); Analyse-it Method Evaluation Edition software, version 2.26 (Analyse-it Software Ltd., Leeds, UK); MedCalc software, version 19.8 (MedCalc Software Ltd., Ostend, Belgium); and the moonBook package in R (http://web-r.org/).

## 3 Results

### 3.1 Clinical and demographic information

A total of 220 samples (n = 110 for endocervix and n = 110 for exocervix) from pregnant women were analyzed (**Supplementary Table S2**). The study population consisted of 63 patients with cervical insufficiency receiving cerclage, 21 undergoing prophylactic cerclage, and 26 women with normal pregnancies. Membrane bulging was observed in 41 (37.3%) patients and the median gestational age at cerclage was 22.0 weeks (interquartile range = 5.0 weeks). Among participants with complete clinical information on their outcomes (n = 84; 76.4%), 13 (15.5%) of them exhibited preterm birth. Patients outcomes are presented in **Supplementary Table S3**.

### 3.2 Principal component analysis (PCA)

**Figure 1:**
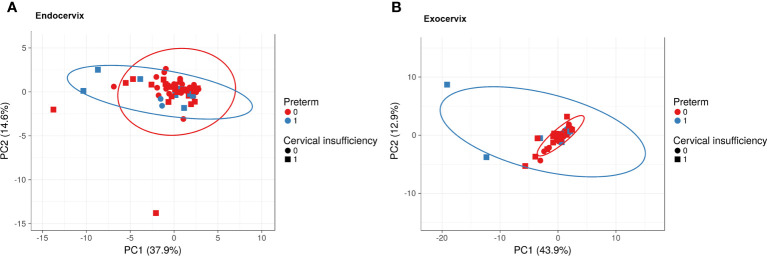
Principal component analysis plots for soluble immune-checkpoint proteins and inflammatory cytokines according to sampling location, cervical insufficiency, and preterm birth. **(A)** Endocervical samples; **(B)** Exocervical samples. Prediction ellipses illustrated as red and blue lines represent probabilities of 0.95.

To investigate the overall profiles, including soluble immune-checkpoint proteins and inflammatory cytokines, according to clinical information such as sampling location, cervical insufficiency, and preterm birth, PCA was performed (**Figure 1**). The X and Y axes of the PCA plot for the endocervix samples show principal components (PC) 1 and 2, respectively, which explain 37.9% and 14.6% of the total variance, respectively (**Figure 1A**). Meanwhile, PCA of the exocervix samples showed that PC1 accounted for 43.9% of the variance and PC2 accounted for 12.9% of the variance in the dataset (**Figure 1B**). Samples from patients exhibiting preterm birth varied more than those who did not and had a similar pattern to those for cervical insufficiency. These patterns were distinct in exocervical samples. PCA loading values for soluble immune-checkpoint proteins and inflammatory cytokines are presented in **Supplementary Table S4** (endocervix) and **Table S5** (exocervix).

### 3.3 Microbiomes, immune-checkpoint proteins, and cytokines according to sampling location

**Figure 2:**
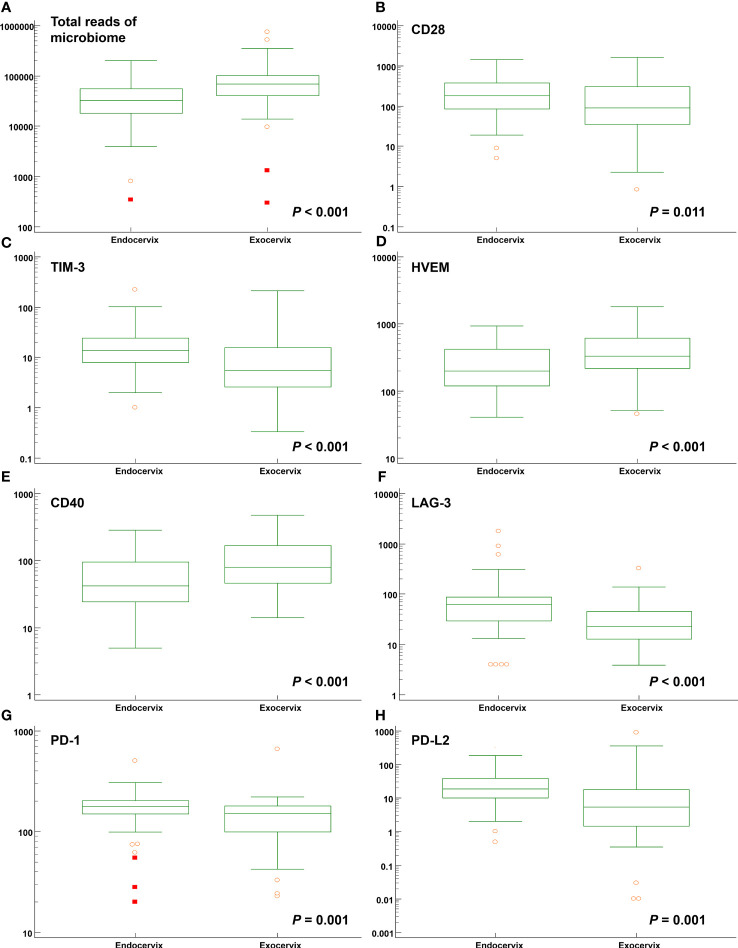
Distribution of total microbiome reads and levels of soluble immune-checkpoint proteins according to sampling locations such as the endocervix and exocervix. Plots for **(A)** total reads; **(B)** CD28; **(C)** TIM-3; **(D)** HVEM; **(E)** CD40; **(F)** LAG-3; **(G)** PD-1; and **(H)** PD-L2.

Comparisons between endocervix and exocervix samples were conducted (**Supplementary Table S6**). After taxonomic profiling of the microbiomes, clinically significant bacteria in the cervical environment, such as *Lactobacillus* spp., *Gardnerella vaginalis*, *Moraxella osloensis*, *Veillonella atypica*, *V. parvula*, *Streptococcus dysgalactiae*, *Ureaplasma urealyticum*, *U. parvum*, *Fusobacterium nucleatum*, *Mycoplasma hominis*, *Sneathia amnii*, *Prevotella enoeca*, *P. fusca*, *P. scopos*, *P. jejuni*, *Megasphaera elsdenii*, and *Megasphaera stantonii* were included in analyses. The median total microbiome reads (68577.5 versus 32169.0) and *Lactobacillus* spp. (41178.5 versus 21109.5) in exocervix samples were two-fold greater than in endocervix samples (**Figure 2A**). However, there were no significant differences at the species level. Among immune-checkpoint proteins, CD 28, TIM-3, LAG-3, PD-1, and PD-L2 showed significantly increased levels in the endocervix than the exocervix. Meanwhile, HVEM and CD40 were present at lower levels in the endocervix (**Figures 2B–H**). Regarding inflammatory cytokines, the endocervix had significantly increased median levels of CCL2, CCL3, CCL4, IL-6, and TNF-α but significantly decreased IL-1α levels (**Supplementary Table S6**). Taken together, the endo- and exocervical microenvironments showed significant differences in their microbiomes, soluble immune-checkpoint proteins, and inflammatory cytokines.

### 3.4 Immune-checkpoint proteins and cytokines in patients with cervical insufficiency

**Figure 3:**
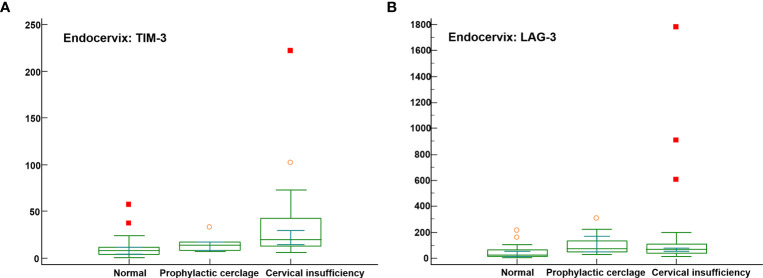
Distribution of soluble immune-checkpoint protein levels in the cervical insufficiency, prophylactic cerclage, and normal pregnant women groups. Plots for **(A)** Endocervical TIM-3; and **(B)** Endocervical LAG-3.

Significant differences were not observed among the endocervical microbiomes of patients undergoing cerclage and prophylactic, cerclage and normal individuals (**Supplementary Table S7**). The levels of five immune-checkpoint proteins, TIM-3, LAG-3, TLR2, PD-L2, and ICOS revealed significant differences. Among them, TIM-3 and LAG-3 with *P*-values less than 0.001 are illustrated in **Figures 3A, B**. For inflammatory cytokines in endocervix, the CCL2, CCL3, CCL4, GM-CSF, IFN-γ, IL-1β, IL-4, IL-6, and IL-13 were significantly different among included groups (**Supplementary Table S7**).

For exocervical samples, the highest total microbiome reads were observed in patients with cervical insufficiency undergoing cerclages (75822.0; *P* = 0.002). Four immune-checkpoint proteins, TIM-3, LAG-3, PD-1, and CD86/B7-2, showed differences. For the inflammatory cytokines, CCL2, CCL3, CCL4, GM-CSF, IFN-γ, IL-4, IL-6, IL-13, and TNF-α were significantly different (**Supplementary Table S8**).

### 3.5 Microbiomes, immune-checkpoint proteins, and cytokines in patients with preterm birth

**Figure 4:**
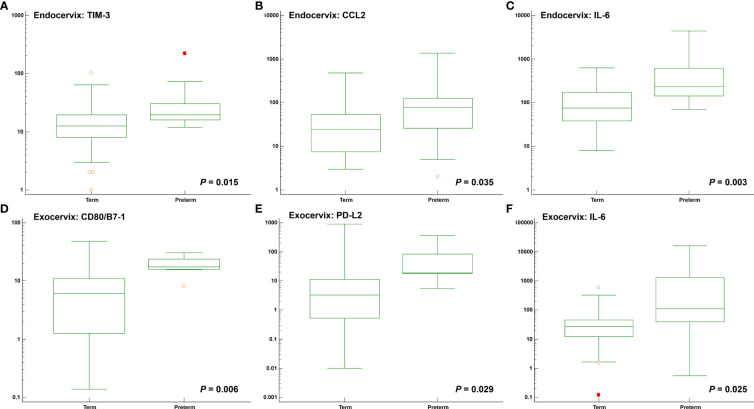
Distribution of soluble immune-checkpoint protein and inflammatory cytokine levels between participants with preterm and term birth. Plots for **(A)** Endocervical TIM-3; **(B)** Endocervical CCL2; **(C)** Endocervical IL-6; **(D)** Exocervical CD80/B7-1; **(E)** Exocervical PD-L2; and **(F)** Exocervical IL-6.

Among clinical characteristics, membrane bulging was significantly different between patients with preterm birth and term participants (*P* = 0.015). Age, gestational age at sampling, and body mass index did not reveal differences. The outcomes for normal participants and patients receiving cerclages with uniconcave balloons were not different (**Supplementary Table S9**). In the microbiomes, *Moraxella osloensis*, *Veillonella atypica*, and *V. parvula* were significantly increased in patients with preterm birth. The median levels of the TIM-3 immune-checkpoint protein as well as the cytokines CCL2, IL-6, and IL-17A were increased in the preterm group compared to the term group (**Figures 4A–C** and **Supplementary Table S10**). For exocervical samples, the median levels of CD80/B7-1, PD-L2, and IL-6 (**Figures 4D–F** and **Supplementary Table S11**) were significantly raised in women with preterm birth. In addition, analyses for patients undergoing cerclage were performed. For the endocervical samples, *V. atypica*, *V. parvula*, and *S. dysgalactiae* were significantly found in patients with preterm birth (**Supplementary Table S12**). Among them, only *S. dysgalactiae* showed significant association with preterm birth (odds ratio [OR] = 14.571; *P* = 0.029). In terms of exocervical samples, *V. atypica* and CD80/B7-1 revealed significant differences (**Supplementary Table S13**).

**Figure 5:**
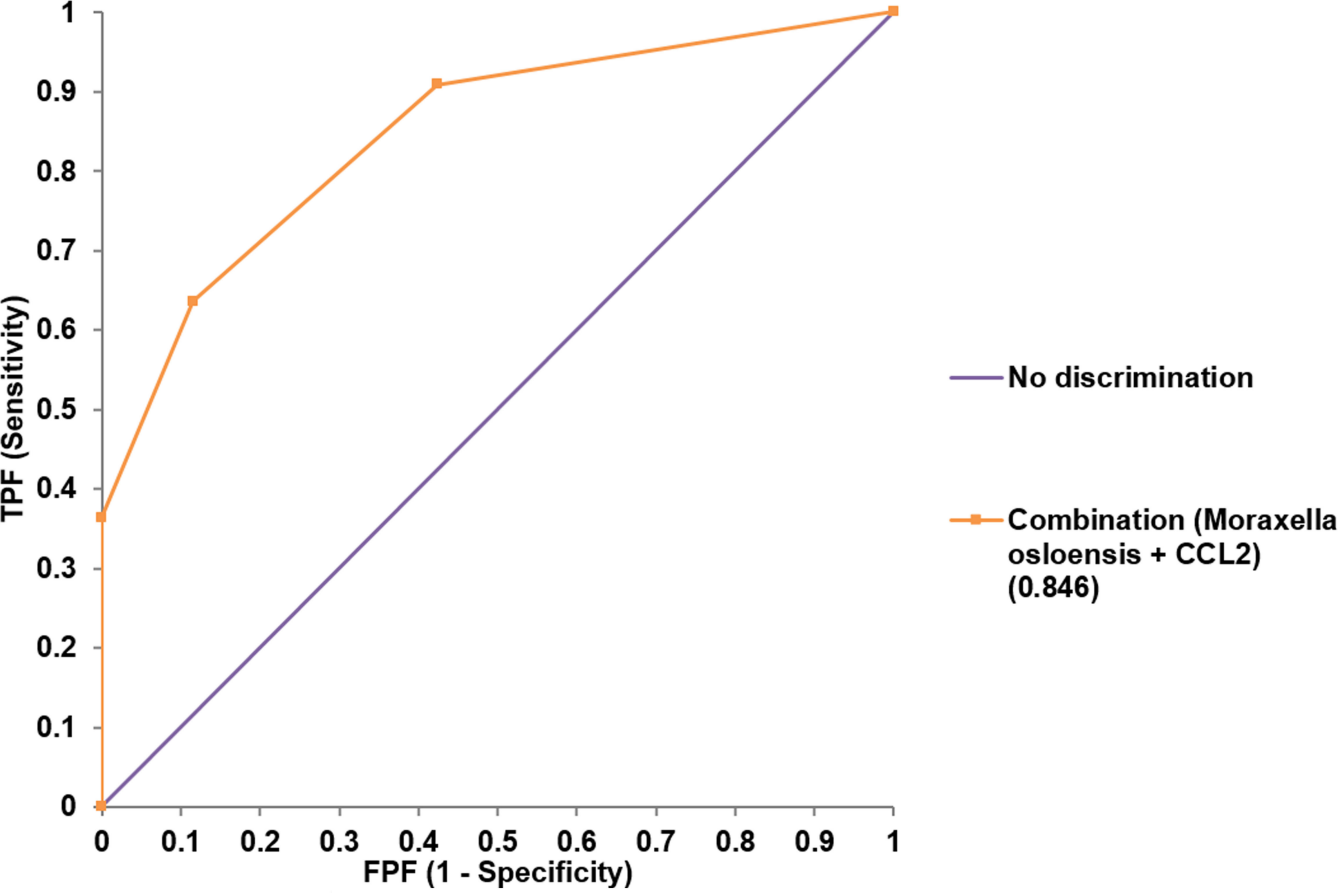
Receiver operating characteristic analysis for the performance of combined variables (*Moraxella osloensis* and CCL2 from endocervical samples) for predicting preterm delivery.

Both univariate and multivariate binary logistic regression analyses were applied to identify variables that correlated independently with the preterm state (**Table 1**). After univariate analyses, variables significantly associated with preterm birth were included in multivariate analyses. The presence of membrane bulging, *Moraxella osloensis*, TIM-3, CCL2, and IL-6 from endocervix were used as predictors. For exocervix, CD80/B7-1, PD-L2, and IL-6 were included as confounding variables for the multivariate analysis. The presence of *M. osloensis* (OR = 14.785; *P* = 0.037) and CCL2 levels higher than 73 pg/mL (OR = 40.049; *P* = 0.005) in the endocervical samples were associated with preterm birth. When factors independently related to preterm birth after multivariate analyses were subjected to ROC analysis, the AUC was 0.846 (95% confidence interval [CI] = 0.733-0.925) (**Figure 5**).

**Table 1:** Univariate and multivariate analyses for predicting preterm birth.

Variable	Univariate		Multivariate*^a^*	
	OR (95% CI)	*P*	OR (95% CI)	*P*
Clinical features				
Age	1.059 (0.909-1.233)	0.464		
Gestational age at sampling	0.997 (0.856-1.161)	0.968		
Body mass index	1.005 (0.879-1.149)	0.946		
Cervical insufficiency	3.429 (0.870-13.514)	0.078		
Membrane bulging	5.357 (1.485-19.333)	0.010	8.915 (0.950-83.667)	0.056
Endocervix				
Microbiota				
* Moraxella osloensis*	4.696 (1.308-16.862)	0.018	14.785 (1.173-186.389)	0.037
Immune-checkpoint protein*^b^*				
TIM-3	11.348 (1.339-96.188)	0.030	9.387 (0.157-561.688)	0.283
Inflammatory cytokine*^b^*				
CCL2	13.417 (3.011-59.789)	0.001	40.049 (3.040-527.575)	0.005
IL-6	9.000 (1.715-47.223)	0.009	1.204 (0.073-19.933)	0.897
Exocervix				
Immune-checkpoint protein*^b^*				
CD80/B7-1	31.500 (2.940-337.558)	0.004	20.314 (1.021-404.347)	0.049
PD-L2	18.000 (1.692-191.529)	0.017	13.680 (0.544-344.046)	0.112
Inflammatory cytokine*^b^*				
IL-6	7.636 (1.684-34.627)	0.008	1.139 (0.047-27.856)	0.936

**^a^** Variables with P-values less than 0.05 in univariate analyses were included in multivariate analyses.

^b^ Cutoffs for continuous values for the immune-checkpoint proteins and inflammatory cytokines were estimated using the Youden index.

OR, odds ratio; CI, confidence interval.

### 3.6 Correlations of immune-checkpoint proteins with microbiomes and cytokines

**Figure 6:**
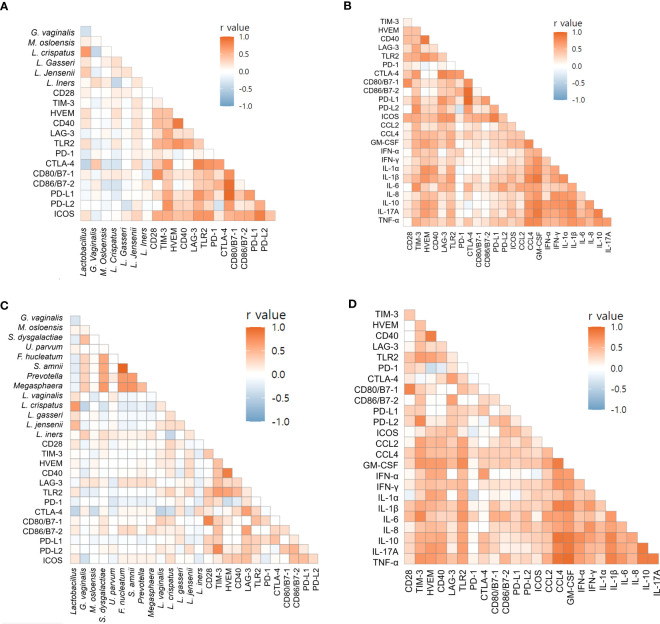
Correlations of soluble immune-checkpoint proteins with microbiomes and inflammatory cytokines based on Spearman’s rank correlation analyses. **(A)** Soluble immune-checkpoint proteins with microbiomes in endocervical samples; **(B)** Soluble immune-checkpoint proteins with inflammatory cytokines in endocervical samples. **(C)** Soluble immune-checkpoint proteins with microbiome in exocervical samples; **(D)** Soluble immune-checkpoint proteins with inflammatory cytokines in exocervical samples.

Using Spearman’s correlation coefficients, we focused on markers associated with preterm birth. For endocervix, *M. osloensis* was negatively correlated with TIM-3, CD80/B7-1, and PD-L2 (**Figure 6A**, **Supplementary Table S14**). Among the immune-checkpoint proteins, PD-L2 showed strong correlation with TIM-3 and CD80/B7-1. Among inflammatory cytokines, CCL2 and IL-6 showed no significant correlation with immune-checkpoint proteins (**Figure 6B**, **Supplementary Table S15**). In exocervical samples, CD80/B7-1 was positively correlated with CD28 and PD-1 (**Figure 6C**, **Supplementary Table S16**). PD-L2 also showed positive correlation with TIM-3. Similar to the endocervix, IL-6 in exocervical samples did not show significant correlation with any immune-checkpoint proteins (**Figure 6D**, **Supplementary Table S17**).

## 4 Discussion

We have shown that cervical immune-checkpoint proteins can be measured indicating the presence of their soluble forms in the cervical microenvironment. For cervical locations, total microbiome and *Lactobacillus* reads, seven immune-checkpoint proteins, and six inflammatory cytokines were different between endo- and exocervical samples. No significant differences were found in microbiome distributions among patients undergoing cerclage, patients undergoing prophylactic cerclage, and normal individuals for endocervical samples. Meanwhile, five immune-checkpoint proteins and nine inflammatory cytokines were different. For exocervical samples, total microbiome reads, four immune-checkpoint proteins, and nine inflammatory cytokines showed differences. For patients with preterm birth, two species, one immune-checkpoint protein, and three inflammatory cytokines from endocervical samples, as well as two immune-checkpoint proteins and one inflammatory cytokine from exocervical samples, were significantly different. The area under the ROC curve for the combined presence of *M. osloensis* and CCL2 was 0.846, suggesting excellent performance for predicting preterm birth.

Soluble immune-checkpoint proteins, which may function differently from their membrane-bound forms, have been measured in serum (19) and cervicovaginal lavages (20). However, their role in cervical insufficiency has not been examined. We found that they can be measured using minimally invasive procedures. Furthermore, our data showed differences between endocervix and exocervix in patients with cervical insufficiency. Other microbiome comparisons between vagina and cervical canal have been conducted in women with endometriosis (21) and pregnant women (22). However, there were no data for the comparison between endo- and exocervical samples in cervical insufficiency providing information about precise sampling locations. The locations evidently had an effect on microbiomes because only the exocervix, which is nearer to cervical canal and vagina, showed differences between cervical insufficiency and normal pregnancy.

Microbiome studies on patients with cervical insufficiency are rare. Our data showed non-significant differences in microbiomes, except for exocervical samples. A previous study reported no bacterial composition differences between vagina and cervical canal in patients with cervical insufficiency, suggesting that cervical incompetence enhances exchange between these communities (22). Another study has shown differences between these communities owing to cervical obstruction in normal pregnancies (23).

Inflammatory cytokines were measured in cervicovaginal fluid in a previous study on cervical insufficiency (24). The study reported higher levels of IL-6, in agreement with our data from both the endo- and exocervical samples. Vaginal levels of IL-6 have been reported to be predictive (24, 25), consistent with our results. Data for immune-checkpoint proteins in cervical insufficiency have yet to be reported, to the best of our knowledge.

Preterm delivery is an important complication associated with cervical insufficiency (3, 24). Reduced abundance of *Lactobacillus* spp. has been correlated with premature cervical dilation, whereas *G. vaginalis* has been associated with unsuccessful rescue cerclage (3). The dominance of *L. iners* with *Lactobacillus* depletion was observed in women with cervical shortening, which often precedes preterm delivery (26). A network meta-analysis showed that women with low *Lactobacillus* spp. abundance were at increased risk (OR = 1.69) for preterm birth when compared to those with *L. crispatus* dominance (27). There are multiple reports of dysbiotic conditions in the vaginal microbiome, such as the emergence of *F. nucleatum*, *M. hominis, Ureaplasma* spp., *Sneathia* spp., *Prevotella* spp., and *Megasphaera* spp (20, 28, 29). With respect to the association of *M. osloensis* with preterm birth in our study, it has also been detected in the uterine microbiome in amniotic fluid (30, 31), which is close to the endocervix. In addition, septicemia in a preterm infant, neonatal early-onset sepsis, and ophthalmia caused by *M. osloensis* have been reported (32–34). Considering we used detection through sequencing and a relatively low study population, further studies to validate this species as a predictor of preterm birth are necessary because few studies on the uterine and cervical microbiome have been conducted (28).

Immune-checkpoint proteins are involved in the modulation of T cells (35). Regulatory T cells promote maternal-fetal tolerance, as well as fetal development, throughout gestation (36). Moreover, regulatory T cells express diverse immune-checkpoint molecules involved in immunosuppression, which is critical for effective immune intervention. Several immune-checkpoint proteins, including PD-1, TIM-3, and LAG-3, influence both decidual and peripheral regulatory T cells during pregnancy. According to our data, TIM-3 from endocervical samples was associated with preterm birth. The expression of TIM-3 downregulates signals inhibiting Th1 responses and apoptosis of antigen-specific cells (37). TIM-3 expression dysregulation has been associated with excessive or inhibited inflammatory responses, leading to autoimmune disease and pregnancy complications, including both preterm birth and recurrent spontaneous abortion (38). Thus, the TIM-3 pathway may serve as a potential target for immunotherapeutic approaches (39).

CD80, as a ligand of CTLA-4, is involved in regulating T-cell proliferation and differentiation (40). It was associated with preterm delivery in our exocervical samples. Consistent with our results, alterations in the maternal immune system, such as CD80, have been associated with preterm delivery (41). PD-L2, also associated with preterm delivery in exocervical samples, is another ligand for PD-1; innate immune activators and signaling downstream of cytokine receptors modulate its expression. PD-L2 mainly plays a role in the induction of Th2-driven T-cell immunity (42). Our understanding of PD-L2 is limited, necessitating more studies on this molecule. In addition, the strong correlation among microbiome and immune-checkpoint proteins found in our data has been consistently reported in other diseases (20, 43, 44).

Vaginal levels of the inflammatory cytokine IL-6 have been reported to be predictive in patients with cervical insufficiency (24, 25, 45), consistent with our results. CCL2 is a pro-inflammatory cytokine that is upregulated in the myometrium during labor (46). Abnormal levels of CCL2 have been associated with adverse outcomes such as spontaneous abortion, preeclampsia, and preterm labor (47). A previous study demonstrated that respondents with recurrent pregnancy loss expressed higher level of CCL2 than normal pregnant women (48). CCL2 levels were well above the normal range in plasma and placenta samples from patients with preeclampsia (49). CCL2 also integrates both mechanical and endocrine signals that influence preterm delivery, showing promise as a potential target for therapeutic prevention of preterm birth. Exploring and combining microbiomes, immune-checkpoint proteins, and inflammatory cytokines exhibited excellent performance for predicting preterm birth, supporting comprehensive approaches.

To the best of our knowledge, data for immune-checkpoint proteins in cervical insufficiency and preterm birth have yet to be reported. We found that immune-checkpoint proteins could be measured in both endo- and exocervical microenvironments and showed differences. Potential biomarkers for predicting preterm birth with precise sampling locations were identified for clinical settings. In addition, measuring soluble markers is beneficial because of easy accessibility and non-invasiveness. Limitations of our study include its relatively small sample size. Future studies with larger study populations are needed to validate and extend our findings. Furthermore, we were not able to measure the membrane-bound forms of studied markers, as tissues from normal participants and pregnant patients with cervical insufficiency were not available.

In conclusion, we showed that soluble immune-checkpoint proteins and inflammatory cytokines, as well as microbiomes, can be detected in the endo- and exocervical microenvironments, especially in patients with cervical insufficiency. Their distributions revealed differences between endo- and exocervical microenvironments. Significantly different levels of these markers were found in cervical insufficiency. These patterns were also observed in patients with preterm deliveries. For predicting preterm birth, combining *M. osloensis* and CCL2 from endocervical samples exhibited excellent performance, showing potential as a prognostic biomarker. Understanding the relationships among microbiomes, immune-checkpoint proteins, and inflammatory cytokines has potential in the development of novel strategies for the management of cervical insufficiency and preterm states based on risk stratification.

## Data availability statement

The data presented in the study are deposited in the NCBI Sequence Read Archive, accession number PRJNA836344 and HARVARD Dataverse, accession number https://doi.org/10.7910/DVN/4YF2FU.

## Ethics statement

The studies involving human participants were reviewed and approved by Institutional Review Board of Kangnam Sacred Heart Hospital (HKS 2019-11-024 and 2022-06-010). The patients/participants provided their written informed consent to participate in this study.

## Author contributions

SJ and K-YL conceived the study. WC and YJ analyzed the microbiome data. SC and K-YL led the sampling and clinical data generation. SJ, NL, and KJ generated immunologic data and performed the analyses. SJ wrote the manuscript. M-JP, WS, and K-YL reviewed and edited the manuscript. All authors contributed to the manuscript and approved the submitted version.
