# Supplementary material for: Immune-checkpoint proteins, cytokines, and microbiome impact on patients with cervical insufficiency and preterm birth
Source: Front Immunol. 2023 Jul 24;14:1228647. doi: 10.3389/fimmu.2023.1228647 (PMC10404982; doi:10.3389/fimmu.2023.1228647)
Supplement: Supplementary file 1 [file DataSheet_1.docx]

Supplementary Material

Immune-checkpoint proteins, cytokines, and microbiome impact on patients with cervical insufficiency and preterm birth

Seri Jeong^1^, Won Kyong Cho^2^, Yeonhwa Jo^2^, Soo-Ran Choi^3^, Nuri Lee^1^, Kibum Jeon^4^, Min-Jeong Park^1^, Wonkeun Song^1^, Keun-Young Lee^5*^

^1^Department of Laboratory Medicine, Kangnam Sacred Heart Hospital, Hallym University College of Medicine, Seoul, South Korea

^2^College of Biotechnology and Bioengineering, Sungkyunkwan University, Suwon, South Korea ^3^Department of Obstetrics and Gynecology, Inha University College of Medicine, Inha University Hospital, Incheon, South Korea

^4^Department of Laboratory Medicine, Hangang Sacred Heart Hospital, Hallym University College of Medicine, Seoul, South Korea

^5^ Division of Maternal-Fetal Medicine, Department of Obstetrics and Gynecology, Kangnam Sacred Heart Hospital, Hallym University College of Medicine, Seoul, South Korea

*** Correspondence:**

Keun-Young Lee
[mfmlee@hallym.ac.kr](mailto:mfmlee@hallym.ac.kr)

**
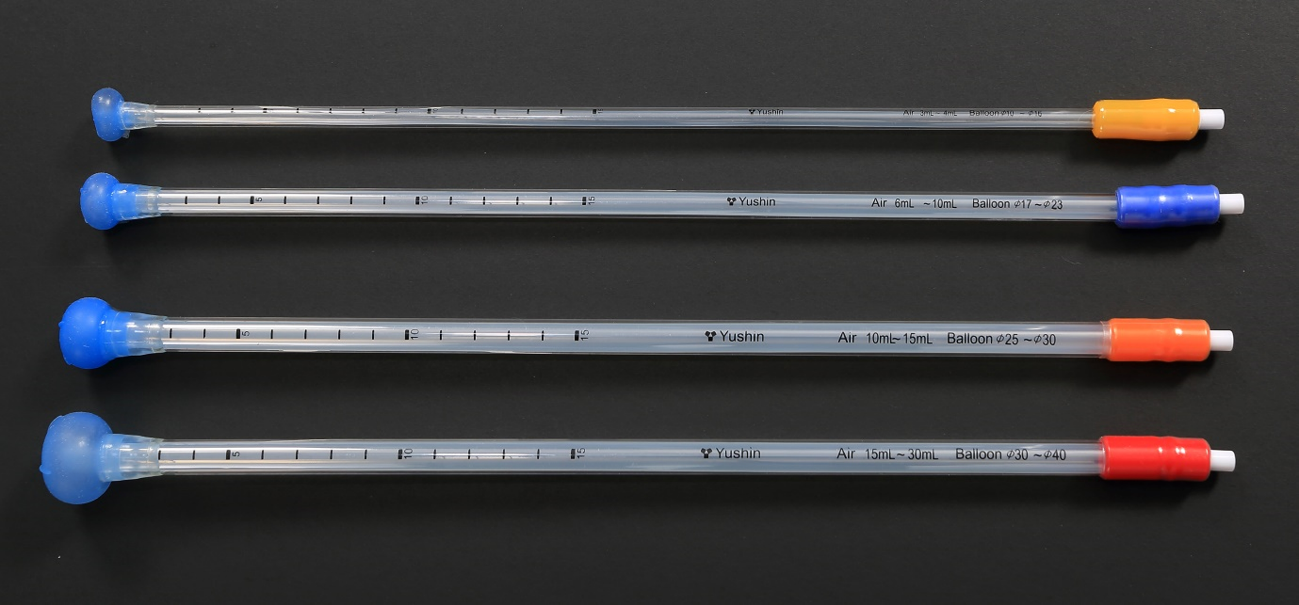
**

**Supplementary Figure S1.** Four different balloons for performing cerclage

**Supplementary Table S1.** The distribution of continuous variables

| Variable | Mean | Mean SE | SD | Skewness | Kurtosis | 1st quartile | Median | 3rd quartile | *P*-value |
| --- | --- | --- | --- | --- | --- | --- | --- | --- | --- |
| Clinical characteristics |  |  |  |  |  |  |  |  |  |
| Age (years) | 33.3 | 0.4 | 4.0 | -0.3 | 0.1 | 31.0 | 33.0 | 36.0 | 0.0783 |
| Gestational age  at sampling (weeks) | 21.3 | 0.4 | 4.0 | -0.5 | -0.6 | 19.0 | 22.0 | 25.0 | <0.0001 |
| Body mass index (kg/m^2^) | 25.8 | 0.4 | 4.2 | 0.6 | 0.2 | 23.0 | 25.0 | 28.0 | 0.0305 |
| Endocervix |  |  |  |  |  |  |  |  |  |
| Total microbiome reads | 46232.5 | 4008.9 | 42045.6 | 1.9 | 4.0 | 18102.5 | 32169.0 | 55408.9 | <0.0001 |
| *Lactobacillus* | 27885.4 | 2523.0 | 26461.1 | 1.4 | 2.1 | 7950.8 | 21109.5 | 39184.8 | <0.0001 |
| CD28 | 309.2 | 39.5 | 323.4 | 1.7 | 2.4 | 84.0 | 183.0 | 372.5 | <0.0001 |
| TIM-3 | 23.1 | 3.7 | 30.6 | 4.5 | 25.9 | 8.0 | 14.0 | 24.1 | <0.0001 |
| HVEM | 290.7 | 26.9 | 226.4 | 1.2 | 0.4 | 119.0 | 200.0 | 420.0 | <0.0001 |
| CD40 | 65.7 | 7.0 | 59.3 | 1.7 | 3.1 | 24.2 | 42.0 | 96.0 | <0.0001 |
| LAG-3 | 114.4 | 30.1 | 246.6 | 5.4 | 33.5 | 29.2 | 63.0 | 87.0 | <0.0001 |
| TLR2 | 157.5 | 16.3 | 136.5 | 2.1 | 6.8 | 62.9 | 113.0 | 226.2 | <0.0001 |
| PD-1 | 177.0 | 7.8 | 66.1 | 1.4 | 8.5 | 150.0 | 179.0 | 204.8 | <0.0001 |
| CTLA-4 | 3.6 | 2.0 | 6.8 | 3.0 | 9.3 | 0.4 | 1.1 | 4.3 | <0.0001 |
| CD80/B7-1 | 15.0 | 1.7 | 13.9 | 1.6 | 2.9 | 4.7 | 12.0 | 21.0 | <0.0001 |
| CD86/B7-2 | 22.1 | 8.9 | 48.2 | 4.1 | 19.0 | 4.7 | 6.0 | 18.3 | <0.0001 |
| PD-L1 | 3.9 | 1.6 | 9.4 | 4.3 | 20.5 | 0.0 | 1.0 | 2.0 | <0.0001 |
| PD-L2 | 49.3 | 11.5 | 87.4 | 3.5 | 13.7 | 9.9 | 18.5 | 39.7 | <0.0001 |
| ICOS | 20.2 | 6.3 | 40.8 | 5.8 | 35.3 | 8.0 | 11.0 | 14.2 | <0.0001 |
| CCL2 | 76.1 | 21.7 | 182.9 | 5.7 | 36.5 | 9.0 | 32.0 | 71.2 | <0.0001 |
| CCL3 | 31.4 | 6.1 | 51.8 | 5.6 | 38.1 | 11.0 | 14.0 | 39.5 | <0.0001 |
| CCL4 | 139.4 | 12.5 | 104.7 | 2.3 | 6.4 | 74.0 | 119.0 | 161.3 | <0.0001 |
| CXCL10 | 5.6 | 1.3 | 9.8 | 3.2 | 9.8 | 1.4 | 2.0 | 4.6 | <0.0001 |
| GM-CSF | 8.7 | 1.0 | 8.2 | 2.0 | 5.1 | 4.0 | 6.0 | 11.6 | <0.0001 |
| IFN-α | 2.8 | 0.3 | 2.0 | 0.7 | 0.4 | 1.0 | 2.0 | 4.0 | 0.0052 |
| IFN-γ | 39.7 | 2.0 | 16.5 | 0.6 | -0.1 | 27.0 | 38.0 | 49.8 | 0.0880 |
| IL-1α | 45.0 | 6.2 | 51.9 | 2.9 | 11.7 | 13.8 | 28.5 | 53.0 | <0.0001 |
| IL-1β | 328.9 | 42.1 | 354.5 | 1.8 | 3.2 | 77.3 | 243.0 | 409.7 | <0.0001 |
| IL-4 | 2.1 | 0.4 | 2.4 | 1.0 | -0.4 | 0.1 | 1.2 | 3.3 | <0.0001 |
| IL-6 | 383.3 | 145.1 | 1213.7 | 5.5 | 32.0 | 48.0 | 96.5 | 240.3 | <0.0001 |
| IL-8 | 893.3 | 223.2 | 1880.4 | 3.8 | 14.6 | 132.8 | 327.0 | 665.5 | <0.0001 |
| IL-10 | 33.6 | 4.1 | 31.6 | 1.6 | 3.1 | 11.8 | 23.5 | 47.8 | <0.0001 |
| IL-12 | 16.7 | 1.7 | 13.4 | 1.3 | 1.0 | 7.0 | 13.0 | 22.1 | <0.0001 |
| IL-13 | 26.1 | 2.9 | 17.6 | 1.0 | 0.2 | 13.7 | 17.0 | 35.7 | 0.0010 |
| IL-17A | 2.5 | 0.3 | 2.1 | 1.6 | 3.4 | 1.0 | 2.0 | 3.0 | <0.0001 |
| TNF-α | 11.3 | 2.1 | 16.2 | 3.2 | 11.5 | 2.0 | 6.0 | 13.0 | <0.0001 |
| Exocervix |  |  |  |  |  |  |  |  |  |
| Total microbiome reads | 91496.5 | 9576.7 | 100441.8 | 3.9 | 19.9 | 40147.2 | 68577.5 | 102688.3 | <0.0001 |
| *Lactobacillus* | 52901.0 | 5291.2 | 55494.3 | 3.0 | 13.1 | 20185.2 | 41178.5 | 68895.5 | <0.0001 |
| CD28 | 216.3 | 44.2 | 321.6 | 3.0 | 9.7 | 34.7 | 90.7 | 303.5 | <0.0001 |
| TIM-3 | 18.7 | 4.9 | 39.2 | 3.8 | 15.0 | 2.6 | 5.5 | 16.0 | <0.0001 |
| HVEM | 435.5 | 37.2 | 311.3 | 1.6 | 4.1 | 217.9 | 331.3 | 613.8 | <0.0001 |
| CD40 | 120.9 | 12.3 | 103.1 | 1.5 | 1.7 | 45.9 | 79.7 | 170.8 | <0.0001 |
| LAG-3 | 38.8 | 6.0 | 47.0 | 4.2 | 23.0 | 12.9 | 23.2 | 45.7 | <0.0001 |
| TLR2 | 140.3 | 12.8 | 103.9 | 0.7 | -0.2 | 52.3 | 124.7 | 203.9 | 0.0047 |
| PD-1 | 144.5 | 10.2 | 85.5 | 3.1 | 19.1 | 98.9 | 152.0 | 181.7 | <0.0001 |
| CTLA-4 | 0.4 | 0.1 | 0.3 | 0.4 | -1.1 | 0.2 | 0.4 | 0.7 | 0.3217 |
| CD80/B7-1 | 11.6 | 1.7 | 10.9 | 1.3 | 2.0 | 2.1 | 8.8 | 18.0 | 0.0023 |
| CD86/B7-2 | 6.7 | 1.2 | 5.7 | 3.3 | 13.3 | 4.2 | 5.5 | 8.0 | <0.0001 |
| PD-L1 | 1.0 | 0.2 | 1.2 | 1.9 | 3.9 | 0.2 | 0.4 | 1.3 | <0.0001 |
| PD-L2 | 64.6 | 29.0 | 171.6 | 3.8 | 16.6 | 1.4 | 5.4 | 18.4 | <0.0001 |
| ICOS | 12.1 | 1.1 | 7.5 | 2.6 | 8.2 | 9.0 | 10.9 | 13.8 | <0.0001 |
| CCL2 | 93.2 | 51.3 | 428.9 | 7.9 | 64.7 | 4.9 | 14.6 | 44.2 | <0.0001 |
| CCL3 | 23.1 | 5.3 | 44.4 | 4.6 | 22.3 | 7.3 | 10.4 | 16.7 | <0.0001 |
| CCL4 | 122.4 | 20.7 | 169.3 | 6.6 | 49.3 | 57.2 | 92.8 | 137.5 | <0.0001 |
| CXCL10 | 5.7 | 2.1 | 16.4 | 7.0 | 52.5 | 1.1 | 2.3 | 4.8 | <0.0001 |
| GM-CSF | 8.3 | 1.3 | 10.8 | 3.8 | 17.4 | 3.3 | 5.4 | 9.3 | <0.0001 |
| IFN-α | 3.2 | 0.3 | 2.2 | 2.0 | 4.8 | 2.0 | 2.5 | 3.9 | <0.0001 |
| IFN-γ | 43.5 | 2.0 | 16.5 | 1.2 | 3.0 | 32.4 | 42.2 | 51.0 | 0.0057 |
| IL-1α | 76.9 | 11.0 | 91.8 | 3.5 | 15.1 | 27.9 | 50.4 | 82.4 | <0.0001 |
| IL-1β | 43.5 | 2.0 | 16.5 | 1.2 | 3.0 | 32.4 | 42.2 | 51.0 | 0.0057 |
| IL-4 | 2.6 | 0.5 | 2.8 | 0.9 | -0.4 | 0.3 | 1.3 | 4.2 | <0.0001 |
| IL-6 | 362.5 | 246.9 | 1990.5 | 7.7 | 61.2 | 12.6 | 33.6 | 73.7 | <0.0001 |
| IL-8 | 627.3 | 152.5 | 1275.7 | 6.9 | 52.9 | 177.8 | 369.6 | 711.6 | <0.0001 |
| IL-10 | 31.4 | 4.8 | 37.3 | 2.1 | 4.2 | 8.1 | 20.0 | 35.7 | <0.0001 |
| IL-12 | 16.9 | 1.8 | 14.1 | 2.0 | 4.3 | 7.8 | 11.6 | 22.1 | <0.0001 |
| IL-13 | 25.1 | 3.6 | 21.7 | 1.5 | 1.7 | 9.3 | 19.3 | 34.6 | <0.0001 |
| IL-17A | 2.5 | 0.3 | 2.6 | 2.2 | 4.9 | 1.0 | 1.5 | 3.2 | <0.0001 |
| TNF-α | 11.4 | 4.7 | 33.2 | 5.5 | 32.3 | 1.6 | 4.0 | 8.3 | <0.0001 |

*SE, standard error; SD, standard deviation.*

**Supplementary Table S2.** Basic characteristics of pregnant women in this study

| Characteristics | Value*^a^* |
| --- | --- |
| Age (years) | 33.0 (31.0-36.0) |
| Body mass index (kg/m^2^) |  |
| < 25 | 54 (49.1%) |
| ≥ 25 | 56 (50.9%) |
| Cervical insufficiency | 63 (57.3%) |
| None-cervical insufficiency | 47 (42.7%) |
| Types of operation |  |
| None | 26 (23.6%) |
| Prophylactic cerclage | 21 (19.1%) |
| Cerclage | 63 (57.3%) |
| Cervical length (mm) | 22.9 (17.4-34.9) |
| Membrane bulging |  |
| No | 69 (62.7%) |
| Yes | 41 (37.3%) |
| Gestational age at cerclage (week) | 22.0 (19.0-24.0) |
| Number of cerclage |  |
| None | 28 (25.5%) |
| Once | 67 (60.9%) |
| Twice | 15 (13.6%) |
| Prior preterm birth |  |
| No | 81 (73.6%) |
| Yes | 29 (26.4%) |
| Glucose level | 89.0 (83.0-95.0) |

*^a^* Data are expressed as median (1st quartile-3rd quartile) or number (%).

**Supplementary Table S3.** The outcomes of pregnant women in this study*^a^*

| Outcome | Normal | Prophylactic cerclage | Cervical insufficiency with cerclage |
| --- | --- | --- | --- |
| Term | 23 (88.5) | 13 (61.9) | 35 (55.6) |
| Preterm | 1 (3.8) | 2 (9.5) | 10 (15.9) |
| Unknown | 2 (7.7) | 6 (28.6) | 18 (28.6) |
| Total | 26 (100.0) | 21 (100.0) | 63 (100.0) |

*^a^* Data are expressed as number (%).

**Supplementary Table S4.** Comparison of microbiome, immune-checkpoint protein, and inflammatory cytokines between endocervix and exocervix samples

| Variable | Endocervix (N = 110) | Exocervix (N = 110) | *P*-value |
| --- | --- | --- | --- |
| Microbiome |  |  |  |
| Total microbiome reads | 32169.0 (18174.0-55230.0) | 68577.5 (40224.0-102365.0) | < 0.001 |
| *Lactobacillus* | 21109.5 (7951.0-39089.0) | 41178.5 (20275.0-68791.0) | < 0.001 |
| *Gardnerella vaginalis* |  |  | 0.275 |
| No | 68 (61.8%) | 59 (53.6%) |  |
| Yes | 42 (38.2%) | 51 (46.4%) |  |
| *Moraxella osloensis* |  |  | 1.000 |
| No | 66 (60.0%) | 66 (60.0%) |  |
| Yes | 44 (40.0%) | 44 (40.0%) |  |
| *Veillonella atypica* |  |  | 1.000 |
| No | 108 (98.2%) | 107 (97.3%) |  |
| Yes | 2 (1.8%) | 3 (2.7%) |  |
| *Veillonella parvula* |  |  | 0.719 |
| No | 107 (97.3%) | 105 (95.5%) |  |
| Yes | 3 (2.7%) | 5 (4.5%) |  |
| *Streptococcus dysgalactiae* |  |  | 0.795 |
| No | 103 (93.6%) | 101 (91.8%) |  |
| Yes | 7 (6.4%) | 9 (8.2%) |  |
| *Ureaplasma urealyticum* |  |  | 0.746 |
| No | 104 (94.5%) | 106 (96.4%) |  |
| Yes | 6 (5.5%) | 4 (3.6%) |  |
| *Ureaplasma parvum* |  |  | 0.414 |
| No | 89 (80.9%) | 83 (75.5%) |  |
| Yes | 21 (19.1%) | 27 (24.5%) |  |
| *Fusobacterium nucleatum* |  |  | 1.000 |
| No | 108 (98.2%) | 107 (97.3%) |  |
| Yes | 2 (1.8%) | 3 (2.7%) |  |
| *Mycoplasma hominis* |  |  | 0.614 |
| No | 109 (99.1%) | 107 (97.3%) |  |
| Yes | 1 (0.9%) | 3 (2.7%) |  |
| *Sneathia amnii* |  |  | 1.000 |
| No | 107 (97.3%) | 107 (97.3%) |  |
| Yes | 3 (2.7%) | 3 (2.7%) |  |
| *Prevotella enoeca* |  |  | 1.000 |
| No | 107 (97.3%) | 107 (97.3%) |  |
| Yes | 3 (2.7%) | 3 (2.7%) |  |
| *Prevotella fusca* |  |  | 1.000 |
| No | 107 (97.3%) | 107 (97.3%) |  |
| Yes | 3 (2.7%) | 3 (2.7%) |  |
| *Prevotella scopos* |  |  | 1.000 |
| No | 107 (97.3%) | 107 (97.3%) |  |
| Yes | 3 (2.7%) | 3 (2.7%) |  |
| *Prevotella jejuni* |  |  | 1.000 |
| No | 107 (97.3%) | 107 (97.3%) |  |
| Yes | 3 (2.7%) | 3 (2.7%) |  |
| *Megasphaera elsdenii* |  |  | 1.000 |
| No | 106 (96.4%) | 105 (95.5%) |  |
| Yes | 4 (3.6%) | 5 (4.5%) |  |
| *Megasphaera stantonii* |  |  | 0.746 |
| No | 106 (96.4%) | 104 (94.5%) |  |
| Yes | 4 (3.6%) | 6 (5.5%) |  |
| *Lactobacillus vaginalis* |  |  | 0.664 |
| No | 77 (70.0%) | 73 (66.4%) |  |
| Yes | 33 (30.0%) | 37 (33.6%) |  |
| *Lactobacillus crispatus* |  |  | 0.881 |
| No | 32 (29.1%) | 30 (27.3%) |  |
| Yes | 78 (70.9%) | 80 (72.7%) |  |
| *Lactobacillus gasseri* |  |  | 1.000 |
| No | 86 (78.2%) | 87 (79.1%) |  |
| Yes | 24 (21.8%) | 23 (20.9%) |  |
| *Lactobacillus jensenii* |  |  | 0.335 |
| No | 70 (63.6%) | 62 (56.4%) |  |
| Yes | 40 (36.4%) | 48 (43.6%) |  |
| *Lactobacillus iners* |  |  | 0.335 |
| No | 48 (43.6%) | 40 (36.4%) |  |
| Yes | 62 (56.4%) | 70 (63.6%) |  |
| Immune checkpoint protein |  |  |  |
| CD28 | 183.0 (84.0-369.5) | 90.7 (34.6-301.6) | 0.011 |
| TIM-3 | 14.0 (8.0-24.0) | 5.5 (2.6-15.9) | < 0.001 |
| HVEM | 200.0 (123.0-406.0) | 331.3 (218.2-612.2) | < 0.001 |
| CD40 | 42.0 (24.5-96.0) | 79.7 (46.0-168.4) | < 0.001 |
| LAG-3 | 63.0 (29.5-87.0) | 23.2 (12.9-45.7) | < 0.001 |
| TLR2 | 113.0 (63.0-226.0) | 124.7 (52.3-203.2) | 0.640 |
| PD-1 | 179.0 (150.0-204.5) | 152.0 (98.9-180.9) | 0.001 |
| CTLA-4 | 1.0 (0.0- 4.5) | 0.3 (0.2- 0.7) | 0.335 |
| CD80/B7-1 | 12.0 (5.0-21.0) | 8.8 (2.3-17.7) | 0.153 |
| CD86/B7-2 | 6.0 (5.0-17.0) | 5.5 (4.3- 8.0) | 0.326 |
| PD-L1 | 1.0 (0.0- 2.0) | 0.4 (0.2- 1.3) | 0.702 |
| PD-L2 | 18.5 (10.0-39.0) | 5.4 (1.7-18.3) | 0.001 |
| ICOS | 11.0 (8.0-14.0) | 10.9 (9.0-13.2) | 0.457 |
| Inflammatory cytokine |  |  |  |
| CCL2 | 32.0 (9.0-69.5) | 14.6 (4.9-43.8) | 0.012 |
| CCL3 | 14.0 (11.0-38.5) | 10.4 (7.3-16.7) | 0.001 |
| CCL4 | 119.0 (74.0-161.0) | 92.8 (57.7-131.0) | 0.031 |
| CXCL10 | 2.0 (1.5- 4.5) | 2.3 (1.1- 4.6) | 0.762 |
| GM-CSF | 6.0 (4.0-11.5) | 5.4 (3.3- 9.2) | 0.407 |
| IFN-α | 2.0 (1.0- 4.0) | 2.5 (1.9- 3.9) | 0.430 |
| IFN-γ | 38.0 (27.0-49.5) | 42.2 (32.5-51.0) | 0.132 |
| IL-1α | 28.5 (14.0-53.0) | 50.4 (28.0-82.0) | 0.001 |
| IL-1β | 243.0 (80.0-409.0) | 111.9 (43.6-418.1) | 0.133 |
| IL-4 | 1.0 (0.0- 3.0) | 1.3 (0.3- 4.0) | 0.115 |
| IL-6 | 96.5 (48.0-240.0) | 33.6 (12.9-73.7) | < 0.001 |
| IL-8 | 327.0 (134.5-652.5) | 369.6 (178.9-702.6) | 0.864 |
| IL-10 | 23.5 (12.0-47.5) | 20.0 (8.2-34.8) | 0.256 |
| IL-12 | 13.0 (7.0-22.0) | 11.6 (7.8-21.6) | 0.905 |
| IL-13 | 17.0 (14.0-35.0) | 19.2 (10.5-33.5) | 0.455 |
| IL-17A | 2.0 (1.0- 3.0) | 1.5 (1.0- 3.1) | 0.314 |
| TNF-α | 6.0 (2.0-13.0) | 4.0 (1.6- 8.0) | 0.038 |

**Supplementary Table S5.** Exocervical principal component analysis loading values

| Variable | PC1 | PC2 | PC3 | PC4 | PC5 | PC6 | PC7 |
| --- | --- | --- | --- | --- | --- | --- | --- |
| CD28 | -0.026125818 | 0.149094931 | -0.390458278 | 0.275710882 | -0.257027989 | 0.228874272 | -0.273755658 |
| TIM-3 | -0.244859414 | -0.064288281 | -0.009766844 | 0.010733143 | 0.018641138 | 0.050021598 | 0.172246754 |
| HVEM | -0.163162495 | 0.302391724 | 0.154636234 | -0.164870734 | -0.363867722 | 0.013177065 | 0.150785677 |
| CD40 | -0.152012536 | 0.233807286 | 0.263022034 | -0.097448758 | -0.328458251 | -0.267912654 | 0.241031405 |
| LAG-3 | -0.054300253 | -0.103736171 | 0.337338149 | 0.568943042 | 0.002615192 | 0.060443302 | 0.084120004 |
| TLR2 | -0.099516696 | 0.19818444 | -0.08656898 | 0.072260117 | -0.476264806 | 0.311791355 | 0.322645219 |
| PD-1 | 0.000365351 | -0.13883381 | -0.362331739 | 0.395884157 | 0.144785924 | -0.195854845 | 0.63951554 |
| CTLA-4 | -0.01227025 | 0.03009224 | 0.024633345 | 0.116845516 | -0.022388764 | -0.155121126 | -0.078483311 |
| CD80/B7-1 | -0.017518293 | 0.088617972 | -0.31371723 | 0.176174925 | -0.141940417 | 0.28667985 | -0.295793247 |
| CD86/B7-2 | -0.01439103 | -0.091922619 | 0.179441644 | 0.274055692 | 0.009319528 | -0.007010636 | -0.130713635 |
| PD-L1 | -0.040591489 | -0.12068779 | 0.165828891 | 0.298394911 | -0.101853951 | -0.067908832 | -0.099235808 |
| PD-L2 | -0.057147281 | -0.081043917 | 0.20873236 | -0.032622409 | -0.158160812 | -0.027797294 | -0.031028549 |
| ICOS | -0.026578537 | -0.017251607 | 0.386386852 | 0.309337697 | -0.102170321 | -0.05397011 | -0.19005548 |
| CCL2 | -0.245385693 | -0.314296865 | -0.030834661 | -0.096096522 | -0.091180415 | -0.00321166 | -0.042816797 |
| CCL3 | -0.273626806 | -0.138996053 | -0.068231218 | -0.02266703 | 0.082588232 | 0.104771897 | 0.116050715 |
| CCL4 | -0.265329125 | -0.204130708 | -0.048049576 | -0.011992187 | -0.064520098 | -0.02857704 | -0.079476715 |
| CXCL10 | -0.221915638 | -0.300913973 | 0.004956298 | -0.081248595 | -0.085967034 | -0.040867376 | -0.073588908 |
| GM-CSF | -0.302660802 | 0.016819519 | -0.009629248 | 0.02902414 | 0.064282123 | 0.043909959 | 0.016288436 |
| IFN-α | -0.227622056 | 0.086232533 | -0.114909451 | -0.037354056 | -0.00235791 | -0.30704977 | 0.019452225 |
| IFN-γ | -0.238284698 | 0.082713294 | 0.058915265 | 0.004806645 | 0.086339124 | 0.355744165 | 0.065557462 |
| IL-1α | -0.200855816 | 0.173316724 | 0.245641885 | -0.144080688 | 0.287791149 | 0.200639488 | -0.00208217 |
| IL-1β | -0.176383745 | 0.302904219 | 0.097152756 | 0.083546313 | 0.412662851 | 0.238991437 | 0.021188679 |
| IL-4 | 0.018670258 | 0.031359261 | 0.076872972 | -0.026132679 | -0.000594015 | -0.004759032 | 0.015459301 |
| IL-6 | -0.240071221 | -0.307550158 | -0.042098959 | -0.087115822 | -0.070526817 | -0.008490997 | -0.07377209 |
| IL-8 | -0.259031285 | -0.257557521 | -0.032188718 | -0.074351635 | -0.120974652 | 0.07947182 | -0.075010808 |
| IL-10 | -0.21931604 | 0.250865685 | -0.101146806 | 0.115828965 | 0.042382965 | -0.236764162 | -0.16814967 |
| IL-12 | -0.256106691 | 0.132346713 | -0.006237922 | 0.090432929 | 0.13164632 | 0.056811099 | 0.055514243 |
| IL-13 | -0.1209323 | 0.185632475 | -0.120506301 | 0.040647291 | 0.022244465 | -0.391188006 | -0.163613144 |
| IL-17A | -0.220848929 | 0.236313195 | -0.126092962 | 0.106700666 | 0.003183828 | -0.253029846 | -0.18497515 |
| TNF-α | -0.204804076 | 0.015756007 | -0.079346507 | -0.032119571 | 0.219790519 | -0.054651011 | -0.025542598 |

PC, principal component.

**Supplementary Table S6.** Comparison of microbiome, immune-checkpoint protein, and inflammatory cytokines between endocervix and exocervix samples

| Variable | Endocervix (N = 110) | Exocervix (N = 110) | *P*-value |
| --- | --- | --- | --- |
| Microbiome |  |  |  |
| Total microbiome reads | 32169.0 (18174.0-55230.0) | 68577.5 (40224.0-102365.0) | < 0.001 |
| *Lactobacillus* | 21109.5 (7951.0-39089.0) | 41178.5 (20275.0-68791.0) | < 0.001 |
| *Gardnerella vaginalis* |  |  | 0.275 |
| No | 68 (61.8%) | 59 (53.6%) |  |
| Yes | 42 (38.2%) | 51 (46.4%) |  |
| *Moraxella osloensis* |  |  | 1.000 |
| No | 66 (60.0%) | 66 (60.0%) |  |
| Yes | 44 (40.0%) | 44 (40.0%) |  |
| *Veillonella atypica* |  |  | 1.000 |
| No | 108 (98.2%) | 107 (97.3%) |  |
| Yes | 2 (1.8%) | 3 (2.7%) |  |
| *Veillonella parvula* |  |  | 0.719 |
| No | 107 (97.3%) | 105 (95.5%) |  |
| Yes | 3 (2.7%) | 5 (4.5%) |  |
| *Streptococcus dysgalactiae* |  |  | 0.795 |
| No | 103 (93.6%) | 101 (91.8%) |  |
| Yes | 7 (6.4%) | 9 (8.2%) |  |
| *Ureaplasma urealyticum* |  |  | 0.746 |
| No | 104 (94.5%) | 106 (96.4%) |  |
| Yes | 6 (5.5%) | 4 (3.6%) |  |
| *Ureaplasma parvum* |  |  | 0.414 |
| No | 89 (80.9%) | 83 (75.5%) |  |
| Yes | 21 (19.1%) | 27 (24.5%) |  |
| *Fusobacterium nucleatum* |  |  | 1.000 |
| No | 108 (98.2%) | 107 (97.3%) |  |
| Yes | 2 (1.8%) | 3 (2.7%) |  |
| *Mycoplasma hominis* |  |  | 0.614 |
| No | 109 (99.1%) | 107 (97.3%) |  |
| Yes | 1 (0.9%) | 3 (2.7%) |  |
| *Sneathia amnii* |  |  | 1.000 |
| No | 107 (97.3%) | 107 (97.3%) |  |
| Yes | 3 (2.7%) | 3 (2.7%) |  |
| *Prevotella enoeca* |  |  | 1.000 |
| No | 107 (97.3%) | 107 (97.3%) |  |
| Yes | 3 (2.7%) | 3 (2.7%) |  |
| *Prevotella fusca* |  |  | 1.000 |
| No | 107 (97.3%) | 107 (97.3%) |  |
| Yes | 3 (2.7%) | 3 (2.7%) |  |
| *Prevotella scopos* |  |  | 1.000 |
| No | 107 (97.3%) | 107 (97.3%) |  |
| Yes | 3 (2.7%) | 3 (2.7%) |  |
| *Prevotella jejuni* |  |  | 1.000 |
| No | 107 (97.3%) | 107 (97.3%) |  |
| Yes | 3 (2.7%) | 3 (2.7%) |  |
| *Megasphaera elsdenii* |  |  | 1.000 |
| No | 106 (96.4%) | 105 (95.5%) |  |
| Yes | 4 (3.6%) | 5 (4.5%) |  |
| *Megasphaera stantonii* |  |  | 0.746 |
| No | 106 (96.4%) | 104 (94.5%) |  |
| Yes | 4 (3.6%) | 6 (5.5%) |  |
| *Lactobacillus vaginalis* |  |  | 0.664 |
| No | 77 (70.0%) | 73 (66.4%) |  |
| Yes | 33 (30.0%) | 37 (33.6%) |  |
| *Lactobacillus crispatus* |  |  | 0.881 |
| No | 32 (29.1%) | 30 (27.3%) |  |
| Yes | 78 (70.9%) | 80 (72.7%) |  |
| *Lactobacillus gasseri* |  |  | 1.000 |
| No | 86 (78.2%) | 87 (79.1%) |  |
| Yes | 24 (21.8%) | 23 (20.9%) |  |
| *Lactobacillus jensenii* |  |  | 0.335 |
| No | 70 (63.6%) | 62 (56.4%) |  |
| Yes | 40 (36.4%) | 48 (43.6%) |  |
| *Lactobacillus iners* |  |  | 0.335 |
| No | 48 (43.6%) | 40 (36.4%) |  |
| Yes | 62 (56.4%) | 70 (63.6%) |  |
| Immune checkpoint protein |  |  |  |
| CD28 | 183.0 (84.0-369.5) | 90.7 (34.6-301.6) | 0.011 |
| TIM-3 | 14.0 (8.0-24.0) | 5.5 (2.6-15.9) | < 0.001 |
| HVEM | 200.0 (123.0-406.0) | 331.3 (218.2-612.2) | < 0.001 |
| CD40 | 42.0 (24.5-96.0) | 79.7 (46.0-168.4) | < 0.001 |
| LAG-3 | 63.0 (29.5-87.0) | 23.2 (12.9-45.7) | < 0.001 |
| TLR2 | 113.0 (63.0-226.0) | 124.7 (52.3-203.2) | 0.640 |
| PD-1 | 179.0 (150.0-204.5) | 152.0 (98.9-180.9) | 0.001 |
| CTLA-4 | 1.0 (0.0- 4.5) | 0.3 (0.2- 0.7) | 0.335 |
| CD80/B7-1 | 12.0 (5.0-21.0) | 8.8 (2.3-17.7) | 0.153 |
| CD86/B7-2 | 6.0 (5.0-17.0) | 5.5 (4.3- 8.0) | 0.326 |
| PD-L1 | 1.0 (0.0- 2.0) | 0.4 (0.2- 1.3) | 0.702 |
| PD-L2 | 18.5 (10.0-39.0) | 5.4 (1.7-18.3) | 0.001 |
| ICOS | 11.0 (8.0-14.0) | 10.9 (9.0-13.2) | 0.457 |
| Inflammatory cytokine |  |  |  |
| CCL2 | 32.0 (9.0-69.5) | 14.6 (4.9-43.8) | 0.012 |
| CCL3 | 14.0 (11.0-38.5) | 10.4 (7.3-16.7) | 0.001 |
| CCL4 | 119.0 (74.0-161.0) | 92.8 (57.7-131.0) | 0.031 |
| CXCL10 | 2.0 (1.5- 4.5) | 2.3 (1.1- 4.6) | 0.762 |
| GM-CSF | 6.0 (4.0-11.5) | 5.4 (3.3- 9.2) | 0.407 |
| IFN-α | 2.0 (1.0- 4.0) | 2.5 (1.9- 3.9) | 0.430 |
| IFN-γ | 38.0 (27.0-49.5) | 42.2 (32.5-51.0) | 0.132 |
| IL-1α | 28.5 (14.0-53.0) | 50.4 (28.0-82.0) | 0.001 |
| IL-1β | 243.0 (80.0-409.0) | 111.9 (43.6-418.1) | 0.133 |
| IL-4 | 1.0 (0.0- 3.0) | 1.3 (0.3- 4.0) | 0.115 |
| IL-6 | 96.5 (48.0-240.0) | 33.6 (12.9-73.7) | < 0.001 |
| IL-8 | 327.0 (134.5-652.5) | 369.6 (178.9-702.6) | 0.864 |
| IL-10 | 23.5 (12.0-47.5) | 20.0 (8.2-34.8) | 0.256 |
| IL-12 | 13.0 (7.0-22.0) | 11.6 (7.8-21.6) | 0.905 |
| IL-13 | 17.0 (14.0-35.0) | 19.2 (10.5-33.5) | 0.455 |
| IL-17A | 2.0 (1.0- 3.0) | 1.5 (1.0- 3.1) | 0.314 |
| TNF-α | 6.0 (2.0-13.0) | 4.0 (1.6- 8.0) | 0.038 |

**Supplementary Table S7.** Endocervical comparison of microbiome, immune-checkpoint protein, and inflammatory cytokines among patients undergoing cerclage, propylactic cerclage, and normal individuals

| Variable | Normal (N = 26) | Prophylactic cerclage (N = 21) | Cervical insufficiency with cerclage (N = 63) | *P*-value |
| --- | --- | --- | --- | --- |
| Microbiome |  |  |  |  |
| Total microbiome reads | 22877.5 (14387.0-48291.0) | 27740.0 (19867.0-48670.0) | 37303.0 (24920.5-64069.5) | 0.137 |
| *Lactobacillus* | 19886.0 (13047.0-39089.0) | 22833.0 (9338.0-31784.0) | 22363.0 (5637.5-39034.0) | 0.925 |
| *Gardnerella vaginalis* | |  |  | 0.448 |
| No | 15 (57.7%) | 11 (52.4%) | 42 (66.7%) |  |
| Yes | 11 (42.3%) | 10 (47.6%) | 21 (33.3%) |  |
| *Moraxella osloensis* | |  |  | 0.057 |
| No | 20 (76.9%) | 9 (42.9%) | 37 (58.7%) |  |
| Yes | 6 (23.1%) | 12 (57.1%) | 26 (41.3%) |  |
| *Veillonella atypica* |  |  |  | 1.000 |
| No | 26 (100.0%) | 21 (100.0%) | 61 (96.8%) |  |
| Yes | 0 ( 0.0%) | 0 ( 0.0%) | 2 ( 3.2%) |  |
| *Veillonella parvula* | |  |  | 0.575 |
| No | 26 (100.0%) | 21 (100.0%) | 60 (95.2%) |  |
| Yes | 0 ( 0.0%) | 0 ( 0.0%) | 3 ( 4.8%) |  |
| *Streptococcus dysgalactiae* | |  |  | 0.872 |
| No | 25 (96.2%) | 20 (95.2%) | 58 (92.1%) |  |
| Yes | 1 ( 3.8%) | 1 ( 4.8%) | 5 ( 7.9%) |  |
| *Ureaplasma urealyticum* | |  |  | 0.314 |
| No | 26 (100.0%) | 19 (90.5%) | 59 (93.7%) |  |
| Yes | 0 ( 0.0%) | 2 ( 9.5%) | 4 ( 6.3%) |  |
| *Ureaplasma parvum* | |  |  | 0.894 |
| No | 21 (80.8%) | 18 (85.7%) | 50 (79.4%) |  |
| Yes | 5 (19.2%) | 3 (14.3%) | 13 (20.6%) |  |
| *Fusobacterium nucleatum* | |  |  | 0.401 |
| No | 26 (100.0%) | 20 (95.2%) | 62 (98.4%) |  |
| Yes | 0 ( 0.0%) | 1 ( 4.8%) | 1 ( 1.6%) |  |
| *Mycoplasma hominis* | |  |  | 1.000 |
| No | 26 (100.0%) | 21 (100.0%) | 62 (98.4%) |  |
| Yes | 0 ( 0.0%) | 0 ( 0.0%) | 1 ( 1.6%) |  |
| *Sneathia amnii* |  |  |  | 0.765 |
| No | 26 (100.0%) | 20 (95.2%) | 61 (96.8%) |  |
| Yes | 0 ( 0.0%) | 1 ( 4.8%) | 2 ( 3.2%) |  |
| *Prevotella enoeca* | |  |  | 0.765 |
| No | 26 (100.0%) | 20 (95.2%) | 61 (96.8%) |  |
| Yes | 0 ( 0.0%) | 1 ( 4.8%) | 2 ( 3.2%) |  |
| *Prevotella fusca* |  |  |  | 0.765 |
| No | 26 (100.0%) | 20 (95.2%) | 61 (96.8%) |  |
| Yes | 0 ( 0.0%) | 1 ( 4.8%) | 2 ( 3.2%) |  |
| *Prevotella scopos* |  |  |  | 0.765 |
| No | 26 (100.0%) | 20 (95.2%) | 61 (96.8%) |  |
| Yes | 0 ( 0.0%) | 1 ( 4.8%) | 2 ( 3.2%) |  |
| *Prevotella jejuni* |  |  |  | 0.765 |
| No | 26 (100.0%) | 20 (95.2%) | 61 (96.8%) |  |
| Yes | 0 ( 0.0%) | 1 ( 4.8%) | 2 ( 3.2%) |  |
| *Megasphaera elsdenii* | |  |  | 0.636 |
| No | 26 (100.0%) | 20 (95.2%) | 60 (95.2%) |  |
| Yes | 0 ( 0.0%) | 1 ( 4.8%) | 3 ( 4.8%) |  |
| *Megasphaera stantonii* | |  |  | 0.636 |
| No | 26 (100.0%) | 20 (95.2%) | 60 (95.2%) |  |
| Yes | 0 ( 0.0%) | 1 ( 4.8%) | 3 ( 4.8%) |  |
| *Lactobacillus vaginalis* | |  |  | 0.442 |
| No | 16 (61.5%) | 14 (66.7%) | 47 (74.6%) |  |
| Yes | 10 (38.5%) | 7 (33.3%) | 16 (25.4%) |  |
| *Lactobacillus crispatus* | |  |  | 0.600 |
| No | 7 (26.9%) | 8 (38.1%) | 17 (27.0%) |  |
| Yes | 19 (73.1%) | 13 (61.9%) | 46 (73.0%) |  |
| *Lactobacillus gasseri* | |  |  | 0.633 |
| No | 20 (76.9%) | 15 (71.4%) | 51 (81.0%) |  |
| Yes | 6 (23.1%) | 6 (28.6%) | 12 (19.0%) |  |
| *Lactobacillus jensenii* | |  |  | 0.122 |
| No | 19 (73.1%) | 16 (76.2%) | 35 (55.6%) |  |
| Yes | 7 (26.9%) | 5 (23.8%) | 28 (44.4%) |  |
| *Lactobacillus iners* |  |  |  | 0.571 |
| No | 12 (46.2%) | 11 (52.4%) | 25 (39.7%) |  |
| Yes | 14 (53.8%) | 10 (47.6%) | 38 (60.3%) |  |
| Immune checkpoint protein |  |  |  |  |
| CD28 | 158.0 (80.0-385.0) | 298.0 (133.5-507.5) | 163.0 (104.5-358.0) | 0.699 |
| TIM-3 | 8.0 ( 4.0-12.0) | 14.0 ( 8.5-17.0) | 20.0 (13.5-42.5) | < 0.001 |
| HVEM | 173.0 (104.5-514.0) | 244.5 (121.0-417.5) | 206.0 (146.0-349.5) | 0.762 |
| CD40 | 36.0 (19.5-123.5) | 36.5 (25.0-75.5) | 44.0 (28.5-84.5) | 0.800 |
| LAG-3 | 22.5 (13.0-63.0) | 73.0 (51.0-135.5) | 67.0 (42.5-106.5) | 0.001 |
| TLR2 | 65.0 (48.5-166.5) | 170.0 (68.5-288.0) | 133.0 (96.0-216.0) | 0.042 |
| PD-1 | 177.5 (124.5-203.5) | 169.0 (144.0-195.5) | 188.0 (159.5-209.0) | 0.464 |
| CD80/B7-1 | 5.0 ( 3.5-14.5) | 19.0 ( 6.5-26.5) | 12.0 ( 7.0-22.0) | 0.063 |
| CD86/B7-2 | 36.0 (36.0-36.0) | 23.0 ( 1.5-58.0) | 6.0 ( 5.0- 8.0) | 0.471 |
| PD-L1 | 0.0 ( 0.0- 1.0) | 2.0 ( 2.0-10.0) | 1.0 ( 0.0- 2.0) | 0.206 |
| PD-L2 | 10.0 ( 3.0-21.0) | 17.0 (10.0-24.0) | 23.0 (10.5-64.5) | 0.038 |
| ICOS | 9.0 ( 6.0-12.0) | 48.0 (43.0-53.0) | 12.0 (11.0-16.0) | 0.006 |
| Inflammatory cytokine |  |  |  |  |
| CCL2 | 10.0 ( 5.0-27.5) | 28.0 ( 7.5-63.5) | 58.0 (29.5-75.0) | < 0.001 |
| CCL3 | 12.0 ( 9.0-15.0) | 15.5 (10.5-43.5) | 24.0 (12.0-49.0) | 0.004 |
| CCL4 | 80.5 (51.0-139.5) | 135.0 (101.5-159.0) | 123.0 (89.0-218.0) | 0.026 |
| CXCL10 | 2.0 ( 2.0- 5.0) | 2.0 ( 1.0- 3.0) | 2.0 ( 1.5- 4.0) | 0.507 |
| GM-CSF | 3.0 ( 1.0- 6.0) | 10.5 ( 5.0-12.0) | 8.0 ( 5.0-11.5) | 0.001 |
| IFN-α | 4.0 ( 1.0- 5.0) | 2.0 ( 1.0- 4.0) | 2.0 ( 2.0- 4.0) | 0.503 |
| IFN-γ | 32.0 (24.0-42.5) | 45.5 (38.5-60.0) | 41.0 (28.0-51.0) | 0.020 |
| IL-1α | 27.0 (10.0-54.5) | 28.5 (17.5-57.5) | 30.0 (16.0-50.0) | 0.911 |
| IL-1β | 95.5 (18.5-249.5) | 416.5 (158.5-631.0) | 286.0 (108.5-442.0) | 0.010 |
| IL-4 | 4.5 ( 1.0- 6.0) | 3.0 ( 2.5- 5.0) | 0.0 ( 0.0- 0.0) | < 0.001 |
| IL-6 | 41.0 (21.0-66.5) | 119.0 (68.0-187.5) | 228.0 (80.5-327.0) | < 0.001 |
| IL-8 | 222.5 (110.0-524.0) | 425.0 (353.5-675.0) | 298.0 (129.0-768.5) | 0.203 |
| IL-10 | 21.0 (10.0-56.0) | 34.0 (18.5-41.5) | 23.0 (11.0-38.0) | 0.829 |
| IL-12 | 9.0 ( 5.5-15.0) | 18.0 ( 7.5-29.0) | 13.0 ( 8.5-21.5) | 0.145 |
| IL-13 | 53.0 (47.0-61.0) | 18.5 (17.0-22.0) | 17.0 (11.0-32.0) | 0.004 |
| IL-17A | 1.0 ( 0.5- 3.0) | 3.0 ( 1.0- 3.0) | 2.0 ( 2.0- 3.0) | 0.098 |
| TNF-α | 6.0 ( 2.5- 9.0) | 5.0 ( 2.5- 9.5) | 8.0 ( 2.0-13.5) | 0.524 |

**Supplementary Table S8.** Exocervical comparison of microbiome, immune-checkpoint protein, and inflammatory cytokines among patients undergoing cerclage, propylactic cerclage, and normal individuals

| Variable | Normal (N = 26) | Prophylactic cerclage (N = 21) | Cervical insufficiency with cerclage (N = 63) | *P*-value |
| --- | --- | --- | --- | --- |
| Microbiome |  |  |  |  |
| Total microbiome reads | 46230.5 (30692.0-61420.0) | 69327.0 (46472.0-92709.0) | 75822.0 (49503.5-122369.5) | 0.002 |
| *Lactobacillus* | 35959.0 (20275.0-47176.0) | 45495.0 (30382.0-65623.0) | 48555.0 (14091.5-76903.5) | 0.334 |
| *Gardnerella vaginalis* |  |  |  | 0.113 |
| No | 12 (46.2%) | 8 (38.1%) | 39 (61.9%) |  |
| Yes | 14 (53.8%) | 13 (61.9%) | 24 (38.1%) |  |
| *Moraxella osloensis* |  |  |  | 0.417 |
| No | 17 (65.4%) | 10 (47.6%) | 39 (61.9%) |  |
| Yes | 9 (34.6%) | 11 (52.4%) | 24 (38.1%) |  |
| *Veillonella atypica* |  |  |  | 0.765 |
| No | 26 (100.0%) | 20 (95.2%) | 61 (96.8%) |  |
| Yes | 0 ( 0.0%) | 1 ( 4.8%) | 2 ( 3.2%) |  |
| *Veillonella parvula* |  |  |  | 0.482 |
| No | 26 (100.0%) | 20 (95.2%) | 59 (93.7%) |  |
| Yes | 0 ( 0.0%) | 1 ( 4.8%) | 4 ( 6.3%) |  |
| *Streptococcus dysgalactiae* |  |  |  | 0.444 |
| No | 25 (96.2%) | 18 (85.7%) | 58 (92.1%) |  |
| Yes | 1 ( 3.8%) | 3 (14.3%) | 5 ( 7.9%) |  |
| *Ureaplasma urealyticum* |  |  |  | 0.636 |
| No | 26 (100.0%) | 20 (95.2%) | 60 (95.2%) |  |
| Yes | 0 ( 0.0%) | 1 ( 4.8%) | 3 ( 4.8%) |  |
| *Ureaplasma parvum* |  |  |  | 0.739 |
| No | 21 (80.8%) | 16 (76.2%) | 46 (73.0%) |  |
| Yes | 5 (19.2%) | 5 (23.8%) | 17 (27.0%) |  |
| *Fusobacterium nucleatum* | |  |  | 0.765 |
| No | 26 (100.0%) | 20 (95.2%) | 61 (96.8%) |  |
| Yes | 0 ( 0.0%) | 1 ( 4.8%) | 2 ( 3.2%) |  |
| *Mycoplasma hominis* |  |  |  | 0.765 |
| No | 26 (100.0%) | 20 (95.2%) | 61 (96.8%) |  |
| Yes | 0 ( 0.0%) | 1 ( 4.8%) | 2 ( 3.2%) |  |
| *Sneathia amnii* |  |  |  | 0.765 |
| No | 26 (100.0%) | 20 (95.2%) | 61 (96.8%) |  |
| Yes | 0 ( 0.0%) | 1 ( 4.8%) | 2 ( 3.2%) |  |
| *Prevotella enoeca* |  |  |  | 0.765 |
| No | 26 (100.0%) | 20 (95.2%) | 61 (96.8%) |  |
| Yes | 0 ( 0.0%) | 1 ( 4.8%) | 2 ( 3.2%) |  |
| *Prevotella fusca* |  |  |  | 0.765 |
| No | 26 (100.0%) | 20 (95.2%) | 61 (96.8%) |  |
| Yes | 0 ( 0.0%) | 1 ( 4.8%) | 2 ( 3.2%) |  |
| *Prevotella scopos* |  |  |  | 0.765 |
| No | 26 (100.0%) | 20 (95.2%) | 61 (96.8%) |  |
| Yes | 0 ( 0.0%) | 1 ( 4.8%) | 2 ( 3.2%) |  |
| *Prevotella jejuni* |  |  |  | 0.765 |
| No | 26 (100.0%) | 20 (95.2%) | 61 (96.8%) |  |
| Yes | 0 ( 0.0%) | 1 ( 4.8%) | 2 ( 3.2%) |  |
| *Megasphaera elsdenii* |  |  |  | 0.293 |
| No | 26 (100.0%) | 19 (90.5%) | 60 (95.2%) |  |
| Yes | 0 ( 0.0%) | 2 ( 9.5%) | 3 ( 4.8%) |  |
| *Megasphaera stantonii* |  |  |  | 0.094 |
| No | 26 (100.0%) | 18 (85.7%) | 60 (95.2%) |  |
| Yes | 0 ( 0.0%) | 3 (14.3%) | 3 ( 4.8%) |  |
| *Lactobacillus vaginalis* |  |  |  | 0.196 |
| No | 14 (53.8%) | 13 (61.9%) | 46 (73.0%) |  |
| Yes | 12 (46.2%) | 8 (38.1%) | 17 (27.0%) |  |
| *Lactobacillus crispatus* |  |  |  | 0.865 |
| No | 8 (30.8%) | 6 (28.6%) | 16 (25.4%) |  |
| Yes | 18 (69.2%) | 15 (71.4%) | 47 (74.6%) |  |
| *Lactobacillus gasseri* |  |  |  | 0.499 |
| No | 20 (76.9%) | 15 (71.4%) | 52 (82.5%) |  |
| Yes | 6 (23.1%) | 6 (28.6%) | 11 (17.5%) |  |
| *Lactobacillus jensenii* |  |  |  | 0.099 |
| No | 18 (69.2%) | 14 (66.7%) | 30 (47.6%) |  |
| Yes | 8 (30.8%) | 7 (33.3%) | 33 (52.4%) |  |
| *Lactobacillus iners* |  |  |  | 0.935 |
| No | 10 (38.5%) | 8 (38.1%) | 22 (34.9%) |  |
| Yes | 16 (61.5%) | 13 (61.9%) | 41 (65.1%) |  |
| Immune checkpoint protein |  |  |  |  |
| CD28 | 83.7 (15.9-191.5) | 54.2 (26.0-250.0) | 92.4 (58.7-304.4) | 0.613 |
| TIM-3 | 3.4 ( 1.8- 6.9) | 3.4 ( 2.5- 6.1) | 10.6 ( 4.1-24.5) | 0.035 |
| HVEM | 272.9 (167.2-612.0) | 330.3 (230.4-589.2) | 358.7 (234.1-612.2) | 0.748 |
| CD40 | 68.5 (40.8-164.4) | 70.2 (41.9-103.4) | 95.8 (60.2-229.4) | 0.214 |
| LAG-3 | 12.9 (12.9-25.7) | 26.6 (16.4-33.6) | 45.7 (19.4-58.2) | 0.011 |
| TLR2 | 65.3 (45.5-140.1) | 156.6 (83.0-162.1) | 154.6 (68.2-254.9) | 0.06 |
| PD-1 | 134.4 (76.4-172.3) | 110.7 (73.5-152.7) | 170.3 (132.0-194.5) | 0.013 |
| CD80/B7-1 | 4.5 ( 0.5-11.2) | 17.9 (17.4-18.4) | 9.5 ( 2.8-18.5) | 0.121 |
| CD86/B7-2 | 1.4 ( 0.9- 1.9) | - | 5.6 ( 5.5- 8.0) | 0.024 |
| PD-L1 | 0.7 ( 0.3- 1.5) | 2.5 ( 2.5- 2.5) | 0.3 ( 0.2- 1.3) | 0.316 |
| PD-L2 | 2.9 ( 0.5- 7.3) | - | 6.9 ( 2.3-18.5) | 0.325 |
| ICOS | 9.0 ( 9.0-12.4) | - | 10.9 (10.9-14.0) | 0.309 |
| Inflammatory cytokine |  |  |  |  |
| CCL2 | 7.1 ( 2.6-24.2) | 8.3 ( 3.8-52.2) | 23.1 (10.0-54.9) | 0.02 |
| CCL3 | 8.8 ( 6.3-12.3) | 11.2 ( 8.6-17.6) | 11.3 ( 8.9-32.5) | 0.008 |
| CCL4 | 66.9 (37.7-95.7) | 98.7 (79.9-109.9) | 113.8 (76.9-166.3) | 0.007 |
| CXCL10 | 2.9 ( 1.6- 5.9) | 1.8 ( 0.5- 5.1) | 2.2 ( 1.2- 4.2) | 0.433 |
| GM-CSF | 3.9 ( 0.8- 5.7) | 5.4 ( 3.4- 7.3) | 7.4 ( 4.9-12.9) | 0.001 |
| IFN-α | 3.1 ( 2.0- 4.5) | 2.4 ( 1.9- 2.9) | 2.5 ( 2.1- 3.9) | 0.426 |
| IFN-γ | 32.2 (26.8-42.2) | 52.1 (46.8-55.1) | 42.6 (36.6-50.9) | < 0.001 |
| IL-1α | 41.1 (21.4-72.6) | 72.1 (30.7-111.1) | 55.8 (26.5-79.1) | 0.366 |
| IL-1β | 54.2 (20.8-160.5) | 164.2 (69.1-347.4) | 205.6 (59.0-493.6) | 0.111 |
| IL-4 | 7.1 ( 3.6- 8.1) | 3.3 ( 1.7- 4.3) | 0.2 ( 0.1- 0.6) | < 0.001 |
| IL-6 | 18.0 (12.4-40.0) | 20.9 ( 9.6-51.5) | 47.4 (27.6-128.8) | 0.016 |
| IL-8 | 298.7 (79.2-560.8) | 425.0 (191.5-989.2) | 369.6 (215.1-702.6) | 0.335 |
| IL-10 | 11.5 ( 3.6-23.7) | 21.5 (13.3-23.1) | 26.2 (10.6-46.9) | 0.076 |
| IL-12 | 10.4 ( 6.1-12.2) | 14.8 (10.4-19.1) | 12.3 ( 8.8-26.0) | 0.171 |
| IL-13 | 54.5 (26.6-54.5) | 7.0 ( 7.0-12.8) | 20.0 (10.5-33.5) | 0.022 |
| IL-17A | 1.2 ( 0.7- 1.5) | 1.2 ( 1.0- 1.7) | 2.0 ( 1.1- 3.4) | 0.06 |
| TNF-α | 6.3 ( 3.6-10.6) | 2.2 ( 0.9- 2.5) | 4.9 ( 1.7- 8.8) | 0.049 |

**Supplementary Table S9.** Comparison of clinical characteristics between patients with preterm birth and term individuals

| Variable | Term (N = 71) | Preterm (N = 13) | *P*-value |
| --- | --- | --- | --- |
| Age (years) | 33.0 (31.0-36.0) | 34.0 (32.0-36.0) | 0.538 |
| Gestational age  at sampling (weeks) | 22.0 (20.0-25.0) | 23.0 (21.0-24.0) | 0.881 |
| Membrane bulging | 21 (29.6%) | 9 (69.2%) | 0.015 |
| Body mass index (kg/m^2^) | 26.0 (23.0-29.0) | 27.0 (23.0-28.0) | 0.906 |
| Group |  | | 0.138 |
| Normal | 23 (32.4%) | 1 ( 7.7%) |  |
| Prophylactic cerclage | 13 (18.3%) | 2 (15.4%) |  |
| Cervical insufficiency  receiving cerclage | 35 (49.3%) | 10 (76.9%) |  |

**Supplementary Table S10.** Endocervical comparison of microbiome, immune-checkpoint protein, and inflammatory cytokines between patients with preterm and term individuals

| Variable | Term (N = 71) | Preterm (N = 13) | *P*-value |
| --- | --- | --- | --- |
| Microbiome |  |  |  |
| Total microbiome read | 30114.0 (14568.5-53833.5) | 48670.0 (31358.0-79589.0) | 0.051 |
| *Lactobacillus* | 19929.0 (7950.0-36128.0) | 30319.0 (11715.0-48961.0) | 0.282 |
| *Gardnerella vaginalis* |  |  | 0.928 |
| No | 45 (63.4%) | 9 (69.2%) |  |
| Yes | 26 (36.6%) | 4 (30.8%) |  |
| *Moraxella osloensis* |  |  | 0.028 |
| No | 48 (67.6%) | 4 (30.8%) |  |
| Yes | 23 (32.4%) | 9 (69.2%) |  |
| *Veillonella atypica* |  |  | 0.018 |
| No | 71 (100.0%) | 11 (84.6%) |  |
| Yes | 0 (0.0%) | 2 (15.4%) |  |
| *Veillonella parvula* |  |  | 0.001 |
| No | 71 (100.0%) | 10 (76.9%) |  |
| Yes | 0 (0.0%) | 3 (23.1%) |  |
| *Streptococcus dysgalactiae* |  |  | 0.066 |
| No | 68 (95.8%) | 10 (76.9%) |  |
| Yes | 3 (4.2%) | 3 (23.1%) |  |
| *Ureaplasma urealyticum* |  |  | 1.000 |
| No | 67 (94.4%) | 12 (92.3%) |  |
| Yes | 4 (5.6%) | 1 (7.7%) |  |
| *Ureaplasma parvum* |  |  | 1.000 |
| No | 55 (77.5%) | 10 (76.9%) |  |
| Yes | 16 (22.5%) | 3 (23.1%) |  |
| *Fusobacterium nucleatum* |  |  | 0.706 |
| No | 70 (98.6%) | 12 (92.3%) |  |
| Yes | 1 (1.4%) | 1 (7.7%) |  |
| *Mycoplasma hominis* |  |  | 1.000 |
| No | 70 (98.6%) | 13 (100.0%) |  |
| Yes | 1 (1.4%) | 0 (0.0%) |  |
| *Sneathia amnii* |  |  | 0.954 |
| No | 69 (97.2%) | 12 (92.3%) |  |
| Yes | 2 (2.8%) | 1 (7.7%) |  |
| *Prevotella enoeca* |  |  | 0.954 |
| No | 69 (97.2%) | 12 (92.3%) |  |
| Yes | 2 (2.8%) | 1 (7.7%) |  |
| *Prevotella fusca* |  |  | 0.954 |
| No | 69 (97.2%) | 12 (92.3%) |  |
| Yes | 2 (2.8%) | 1 (7.7%) |  |
| *Prevotella scopos* |  |  | 0.954 |
| No | 69 (97.2%) | 12 (92.3%) |  |
| Yes | 2 (2.8%) | 1 (7.7%) |  |
| *Prevotella jejuni* |  |  | 0.954 |
| No | 69 (97.2%) | 12 (92.3%) |  |
| Yes | 2 (2.8%) | 1 (7.7%) |  |
| *Megasphaera elsdenii* |  |  | 1.000 |
| No | 68 (95.8%) | 12 (92.3%) |  |
| Yes | 3 (4.2%) | 1 (7.7%) |  |
| *Megasphaera stantonii* |  |  | 1.000 |
| No | 68 (95.8%) | 12 (92.3%) |  |
| Yes | 3 (4.2%) | 1 (7.7%) |  |
| *Lactobacillus vaginalis* |  |  | 0.886 |
| No | 50 (70.4%) | 10 (76.9%) |  |
| Yes | 21 (29.6%) | 3 (23.1%) |  |
| *Lactobacillus crispatus* |  |  | 1.000 |
| No | 20 (28.2%) | 4 (30.8%) |  |
| Yes | 51 (71.8%) | 9 (69.2%) |  |
| *Lactobacillus gasseri* |  |  | 0.191 |
| No | 50 (70.4%) | 12 (92.3%) |  |
| Yes | 21 (29.6%) | 1 (7.7%) |  |
| *Lactobacillus jensenii* |  |  | 1.000 |
| No | 43 (60.6%) | 8 (61.5%) |  |
| Yes | 28 (39.4%) | 5 (38.5%) |  |
| *Lactobacillus iners* |  |  | 0.891 |
| No | 32 (45.1%) | 5 (38.5%) |  |
| Yes | 39 (54.9%) | 8 (61.5%) |  |
| Immune checkpoint protein |  |  |  |
| CD28 | 173.0 (82.0-369.5) | 282.0 (69.0-598.5) | 0.592 |
| TIM-3 | 12.5 (8.0-19.5) | 19.5 (16.0-30.0) | 0.015 |
| HVEM | 189.0 (111.5-455.5) | 261.0 (195.0-323.5) | 0.202 |
| CD40 | 35.5 (23.5-105.0) | 62.0 (39.0-82.5) | 0.415 |
| LAG-3 | 51.5 (23.0-87.0) | 71.0 (16.0-84.0) | 0.704 |
| TLR2 | 110.0 (60.0-196.0) | 206.5 (99.0-323.0) | 0.069 |
| PD-1 | 177.5 (146.0-202.5) | 181.0 (170.5-199.0) | 0.738 |
| CD80/B7-1 | 7.0 (4.0-19.5) | 17.5 (12.0-23.0) | 0.093 |
| CD86/B7-2 | 6.0 (4.5-15.0) | 7.0 (7.0-17.0) | 0.315 |
| PD-L1 | 1.0 (0.0- 2.0) | 1.0 (1.0- 4.0) | 0.527 |
| PD-L2 | 15.0 (9.0-28.5) | 20.5 (10.0-47.0) | 0.423 |
| ICOS | 11.0 (8.0-14.0) | 11.0 (9.0-30.0) | 0.457 |
| Inflammatory cytokine |  |  |  |
| CCL2 | 24.0 (7.5-54.5) | 77.0 (32.5-115.5) | 0.035 |
| CCL3 | 13.5 (10.5-23.0) | 37.0 (8.0-72.5) | 0.285 |
| CCL4 | 108.0 (70.5-147.0) | 141.0 (73.5-199.0) | 0.277 |
| CXCL10 | 2.0 (1.0- 4.0) | 3.0 (3.0-10.0) | 0.050 |
| GM-CSF | 5.0 (2.0-10.0) | 9.0 (5.5-18.0) | 0.088 |
| IFN-α | 2.0 (1.0- 4.0) | 2.5 (2.0- 4.0) | 0.687 |
| IFN-γ | 36.0 (27.0-48.0) | 44.0 (36.5-53.0) | 0.105 |
| IL-1α | 29.0 (14.0-47.0) | 25.0 (13.5-90.5) | 0.638 |
| IL-1β | 214.0 (68.5-403.5) | 304.0 (134.0-563.0) | 0.281 |
| IL-4 | 2.0 (1.0- 5.0) | 0.0 (0.0- 3.5) | 0.105 |
| IL-6 | 75.5 (38.0-173.0) | 240.5 (142.0-606.0) | 0.003 |
| IL-8 | 292.0 (134.5-541.0) | 452.0 (172.0-907.5) | 0.324 |
| IL-10 | 23.5 (13.0-38.0) | 31.5 (13.0-61.0) | 0.410 |
| IL-12 | 12.0 (6.0-20.5) | 19.5 (10.0-38.0) | 0.068 |
| IL-13 | 18.5 (12.0-44.0) | 17.0 (17.0-31.0) | 0.555 |
| IL-17A | 2.0 (1.0- 3.0) | 2.5 (2.0- 5.0) | 0.043 |
| TNF-α | 6.0 (2.0-10.5) | 8.0 (2.0-34.0) | 0.213 |

**Supplementary Table S11.** Exocervical comparison of microbiomes, immune-checkpoint proteins, and inflammatory cytokines between patients with preterm and term deliveries

| Variable | Term (N = 71) | Preterm (N = 13) | *P*-value |
| --- | --- | --- | --- |
| Microbiome |  |  |  |
| Total microbiome read | 61420.0 (38320.5-108702.5) | 62194.0 (50063.0-116765.0) | 0.536 |
| *Lactobacillus* | 39820.0 (19736.0-66136.5) | 37175.0 (23652.0-92138.0) | 0.916 |
| *Gardnerella vaginalis* |  |  | 0.965 |
| No | 40 (56.3%) | 8 (61.5%) |  |
| Yes | 31 (43.7%) | 5 (38.5%) |  |
| *Moraxella osloensis* |  |  | 0.139 |
| No | 46 (64.8%) | 5 (38.5%) |  |
| Yes | 25 (35.2%) | 8 (61.5%) |  |
| *Veillonella atypica* |  |  | 0.018 |
| No | 71 (100.0%) | 11 (84.6%) |  |
| Yes | 0 (0.0%) | 2 (15.4%) |  |
| *Veillonella parvula* |  |  | 0.354 |
| No | 68 (95.8%) | 11 (84.6%) |  |
| Yes | 3 (4.2%) | 2 (15.4%) |  |
| *Streptococcus dysgalactiae* |  |  | 0.195 |
| No | 66 (93.0%) | 10 (76.9%) |  |
| Yes | 5 (7.0%) | 3 (23.1%) |  |
| *Ureaplasma urealyticum* |  |  | 1.000 |
| No | 68 (95.8%) | 12 (92.3%) |  |
| Yes | 3 (4.2%) | 1 (7.7%) |  |
| *Ureaplasma parvum* |  |  | 1.000 |
| No | 52 (73.2%) | 10 (76.9%) |  |
| Yes | 19 (26.8%) | 3 (23.1%) |  |
| *Fusobacterium nucleatum* |  |  | 0.954 |
| No | 69 (97.2%) | 12 (92.3%) |  |
| Yes | 2 (2.8%) | 1 (7.7%) |  |
| *Mycoplasma hominis* |  |  | 1.000 |
| No | 69 (97.2%) | 13 (100.0%) |  |
| Yes | 2 (2.8%) | 0 (0.0%) |  |
| *Sneathia amnii* |  |  | 0.954 |
| No | 69 (97.2%) | 12 (92.3%) |  |
| Yes | 2 (2.8%) | 1 (7.7%) |  |
| *Prevotella enoeca* |  |  | 0.954 |
| No | 69 (97.2%) | 12 (92.3%) |  |
| Yes | 2 (2.8%) | 1 (7.7%) |  |
| *Prevotella fusca* |  |  | 0.954 |
| No | 69 (97.2%) | 12 (92.3%) |  |
| Yes | 2 (2.8%) | 1 (7.7%) |  |
| *Prevotella scopos* |  |  | 0.954 |
| No | 69 (97.2%) | 12 (92.3%) |  |
| Yes | 2 (2.8%) | 1 (7.7%) |  |
| *Prevotella jejuni* |  |  | 0.954 |
| No | 69 (97.2%) | 12 (92.3%) |  |
| Yes | 2 (2.8%) | 1 (7.7%) |  |
| *Megasphaera elsdenii* |  |  | 1.000 |
| No | 68 (95.8%) | 12 (92.3%) |  |
| Yes | 3 (4.2%) | 1 (7.7%) |  |
| *Megasphaera stantonii* |  |  | 1.000 |
| No | 68 (95.8%) | 12 (92.3%) |  |
| Yes | 3 (4.2%) | 1 (7.7%) |  |
| *Lactobacillus vaginalis* |  |  | 0.661 |
| No | 47 (66.2%) | 10 (76.9%) |  |
| Yes | 24 (33.8%) | 3 (23.1%) |  |
| *Lactobacillus crispatus* |  |  | 1.000 |
| No | 20 (28.2%) | 4 (30.8%) |  |
| Yes | 51 (71.8%) | 9 (69.2%) |  |
| *Lactobacillus gasseri* |  |  | 0.223 |
| No | 51 (71.8%) | 12 (92.3%) |  |
| Yes | 20 (28.2%) | 1 (7.7%) |  |
| *Lactobacillus jensenii* |  |  | 1.000 |
| No | 39 (54.9%) | 7 (53.8%) |  |
| Yes | 32 (45.1%) | 6 (46.2%) |  |
| *Lactobacillus iners* |  |  | 1.000 |
| No | 25 (35.2%) | 4 (30.8%) |  |
| Yes | 46 (64.8%) | 9 (69.2%) |  |
| Immune checkpoint protein |  |  |  |
| CD28 | 84.8 (22.0-238.6) | 363.1 (111.9-446.3) | 0.068 |
| TIM-3 | 4.4 (2.5-12.4) | 15.7 (3.5-75.2) | 0.121 |
| HVEM | 343.3 (216.5-633.2) | 526.1 (275.6-658.7) | 0.495 |
| CD40 | 88.5 (45.3-197.7) | 80.0 (44.8-174.6) | 0.993 |
| LAG-3 | 26.2 (12.9-44.1) | 23.2 (16.4-52.0) | 0.749 |
| TLR2 | 105.8 (51.2-183.3) | 188.5 (98.7-219.4) | 0.104 |
| PD-1 | 136.6 (87.1-178.5) | 150.4 (124.3-174.6) | 0.719 |
| CD80/B7-1 | 6.2 (1.3-10.9) | 17.4 (16.3-22.5) | 0.006 |
| CD86/B7-2 | 5.5 (3.7- 6.5) | 7.4 (6.2- 9.2) | 0.129 |
| PD-L1 | 0.3 (0.2- 1.2) | 0.4 (0.1- 2.5) | 0.735 |
| PD-L2 | 3.3 (0.6-10.5) | 19.0 (18.1-82.8) | 0.029 |
| ICOS | 10.9 (9.0-14.0) | 10.9 (10.9-12.4) | 0.446 |
| Inflammatory cytokine |  |  |  |
| CCL2 | 12.1 (4.3-30.3) | 23.9 (10.6-158.6) | 0.111 |
| CCL3 | 10.3 (7.2-13.8) | 9.7 (7.1-71.8) | 0.371 |
| CCL4 | 83.4 (56.9-118.7) | 94.3 (43.0-258.5) | 0.585 |
| CXCL10 | 2.3 (1.1- 4.6) | 2.5 (0.6- 8.0) | 0.913 |
| GM-CSF | 5.4 (2.9- 8.0) | 4.9 (3.5-14.1) | 0.525 |
| IFN-α | 2.6 (1.9- 4.0) | 2.4 (2.2- 4.1) | 0.680 |
| IFN-γ | 42.2 (31.1-50.3) | 46.5 (33.8-55.8) | 0.381 |
| IL-1α | 51.0 (30.7-79.4) | 37.8 (23.5-117.4) | 0.985 |
| IL-1β | 103.9 (38.9-347.4) | 151.6 (67.1-533.2) | 0.355 |
| IL-4 | 2.4 (0.6- 4.9) | 1.3 (0.7- 3.3) | 0.685 |
| IL-6 | 27.6 (12.4-45.5) | 124.3 (39.2-1324.5) | 0.025 |
| IL-8 | 297.3 (175.6-560.8) | 490.5 (227.3-1203.1) | 0.176 |
| IL-10 | 16.4 (8.2-31.0) | 17.5 (7.9-52.7) | 0.904 |
| IL-12 | 11.3 (8.2-19.7) | 11.5 (7.8-37.3) | 0.601 |
| IL-13 | 15.1 (7.0-36.8) | 26.8 (14.2-43.3) | 0.269 |
| IL-17A | 1.2 (1.0- 2.5) | 1.4 (1.0- 3.9) | 0.811 |
| TNF-α | 3.6 (1.9- 8.4) | 3.1 (0.6-14.8) | 0.698 |

**Supplementary Table S12.** Comparison of the endocervical microbiome, immune-checkpoint proteins, and inflammatory cytokines in patients with cervical insufficiency according to the presence of preterm birth

| Variable | Term (N = 35) | Preterm (N = 10) | *P*-value |
| --- | --- | --- | --- |
| Microbiome |  |  |  |
| Total microbiome read | 38003.0 (14760.0-58045.0) | 47939.0 (31358.0-123906.0) | 0.189 |
| *Lactobacillus* | 20168.0 (6750.0-36128.0) | 22903.5 (7665.0-48961.0) | 0.712 |
| *Gardnerella vaginalis* |  |  | 0.700 |
| No | 25 (71.4%) | 6 (60.0%) |  |
| Yes | 10 (28.6%) | 4 (40.0%) |  |
| *Moraxella osloensis* |  |  | 0.301 |
| No | 21 (60.0%) | 4 (40.0%) |  |
| Yes | 14 (40.0%) | 6 (60.0%) |  |
| *Veillonella atypica* |  |  | 0.045 |
| No | 35 (100.0%) | 8 (80.0%) |  |
| Yes | 0 ( 0.0%) | 2 (20.0%) |  |
| *Veillonella parvula* |  |  | 0.008 |
| No | 35 (100.0%) | 7 (70.0%) |  |
| Yes | 0 ( 0.0%) | 3 (30.0%) |  |
| *Streptococcus dysgalactiae* |  |  | 0.030 |
| No | 34 (97.1%) | 7 (70.0%) |  |
| Yes | 1 ( 2.9%) | 3 (30.0%) |  |
| *Ureaplasma urealyticum* |  |  | 0.539 |
| No | 33 (94.3%) | 9 (90.0%) |  |
| Yes | 2 ( 5.7%) | 1 (10.0%) |  |
| *Ureaplasma parvum* |  |  | 1.000 |
| No | 26 (74.3%) | 8 (80.0%) |  |
| Yes | 9 (25.7%) | 2 (20.0%) |  |
| *Fusobacterium nucleatum* |  |  | 0.222 |
| No | 35 (100.0%) | 9 (90.0%) |  |
| Yes | 0 ( 0.0%) | 1 (10.0%) |  |
| *Mycoplasma hominis* |  |  | 1.000 |
| No | 34 (97.1%) | 10 (100.0%) |  |
| Yes | 1 ( 2.9%) | 0 ( 0.0%) |  |
| *Sneathia amnii* |  |  | 0.399 |
| No | 34 (97.1%) | 9 (90.0%) |  |
| Yes | 1 ( 2.9%) | 1 (10.0%) |  |
| *Prevotella enoeca* |  |  | 0.399 |
| No | 34 (97.1%) | 9 (90.0%) |  |
| Yes | 1 ( 2.9%) | 1 (10.0%) |  |
| *Prevotella fusca* |  |  | 0.399 |
| No | 34 (97.1%) | 9 (90.0%) |  |
| Yes | 1 ( 2.9%) | 1 (10.0%) |  |
| *Prevotella scopos* |  |  | 0.399 |
| No | 34 (97.1%) | 9 (90.0%) |  |
| Yes | 1 ( 2.9%) | 1 (10.0%) |  |
| *Prevotella jejuni* |  |  | 0.399 |
| No | 34 (97.1%) | 9 (90.0%) |  |
| Yes | 1 ( 2.9%) | 1 (10.0%) |  |
| *Megasphaera elsdenii* |  |  | 0.539 |
| No | 33 (94.3%) | 9 (90.0%) |  |
| Yes | 2 ( 5.7%) | 1 (10.0%) |  |
| *Megasphaera stantonii* |  |  | 0.539 |
| No | 33 (94.3%) | 9 (90.0%) |  |
| Yes | 2 ( 5.7%) | 1 (10.0%) |  |
| *Lactobacillus vaginalis* |  |  | 0.687 |
| No | 27 (77.1%) | 7 (70.0%) |  |
| Yes | 8 (22.9%) | 3 (30.0%) |  |
| *Lactobacillus crispatus* |  |  | 0.687 |
| No | 8 (22.9%) | 3 (30.0%) |  |
| Yes | 27 (77.1%) | 7 (70.0%) |  |
| *Lactobacillus gasseri* |  |  | 0.089 |
| No | 24 (68.6%) | 10 (100.0%) |  |
| Yes | 11 (31.4%) | 0 ( 0.0%) |  |
| *Lactobacillus jensenii* |  |  | 0.722 |
| No | 17 (48.6%) | 6 (60.0%) |  |
| Yes | 18 (51.4%) | 4 (40.0%) |  |
| *Lactobacillus iners* |  |  | 0.716 |
| No | 15 (42.9%) | 3 (30.0%) |  |
| Yes | 20 (57.1%) | 7 (70.0%) |  |
| Immune checkpoint protein |  |  |  |
| CD28 | 163.0 (89.0-369.5) | 262.0 (69.0-439.0) | 0.770 |
| TIM-3 | 19.0 (13.5-41.5) | 25.0 (15.5-51.5) | 0.410 |
| HVEM | 196.0 (146.0-416.5) | 255.5 (178.5-303.0) | 0.621 |
| CD40 | 35.0 (27.0-92.5) | 62.5 (39.0-89.0) | 0.381 |
| LAG-3 | 65.0 (46.0-103.0) | 68.0 (16.0-84.0) | 0.648 |
| TLR2 | 138.0 (102.5-184.5) | 144.5 (82.5-274.5) | 0.811 |
| PD-1 | 188.0 (156.5-199.0) | 185.5 (170.5-211.0) | 0.651 |
| CD80/B7-1 | 10.0 (5.0-34.0) | 15.5 (8.0-20.0) | 0.676 |
| CD86/B7-2 | 6.0 (5.5- 8.0) | 7.0 (6.5-12.0) | 0.508 |
| PD-L1 | 1.0 (0.0- 2.0) | 1.0 (0.5- 2.5) | 0.961 |
| PD-L2 | 17.0 (10.0-70.0) | 27.5 (13.5-106.5) | 0.811 |
| ICOS | 13.0 (11.0-16.0) | 11.0 (9.5-20.5) | 0.787 |
| Inflammatory cytokine |  |  |  |
| CCL2 | 52.0 (27.5-69.0) | 76.5 (32.5-238.5) | 0.254 |
| CCL3 | 16.0 (12.0-32.5) | 49.5 (8.0-108.0) | 0.425 |
| CCL4 | 119.0 (74.0-179.0) | 141.0 (73.5-284.5) | 0.420 |
| CXCL10 | 2.0 (1.0- 4.0) | 3.0 (2.5-13.5) | 0.160 |
| GM-CSF | 8.0 (5.0-10.5) | 8.5 (5.5-25.0) | 0.472 |
| IFN-α | 2.0 (2.0- 4.0) | 3.0 (2.0- 5.0) | 0.549 |
| IFN-γ | 36.0 (28.0-46.0) | 43.5 (36.5-66.0) | 0.110 |
| IL-1α | 30.0 (20.0-41.5) | 22.5 (13.5-110.0) | 0.853 |
| IL-1β | 286.0 (103.0-442.0) | 301.5 (134.0-543.0) | 0.690 |
| IL-6 | 228.0 (62.5-284.5) | 286.0 (109.5-2399.0) | 0.260 |
| IL-8 | 217.0 (129.0-387.5) | 656.0 (172.0-2787.5) | 0.163 |
| IL-10 | 23.0 (13.0-32.0) | 24.5 (12.0-61.0) | 0.683 |
| IL-12 | 13.0 (8.0-20.5) | 16.0 (10.0-42.0) | 0.394 |
| IL-13 | 17.0 (11.0-27.0) | 17.0 (15.5-32.0) | 0.689 |
| TNF-α | 7.0 (3.0-10.5) | 8.0 (2.0-39.0) | 0.520 |

**Supplementary Table S13.** Comparison of the exocervical microbiome, immune-checkpoint proteins, and inflammatory cytokines in patients with cervical insufficiency according to the presence of preterm birth

| Variable | Term (N = 35) | Preterm (N = 10) | *P*-value |
| --- | --- | --- | --- |
| Microbiome |  |  |  |
| Total microbiome read | 77632.0 (45510.0-130784.5) | 61082.0 (50063.0-235751.0) | 0.904 |
| *Lactobacillus* | 48555.0 (20548.0-90033.5) | 34068.0 (11411.0-59831.0) | 0.429 |
| *Gardnerella vaginalis* |  |  | 0.710 |
| No | 24 (68.6%) | 6 (60.0%) |  |
| Yes | 11 (31.4%) | 4 (40.0%) |  |
| *Moraxella osloensis* |  |  | 0.281 |
| No | 22 (62.9%) | 4 (40.0%) |  |
| Yes | 13 (37.1%) | 6 (60.0%) |  |
| *Veillonella atypica* |  |  | 0.045 |
| No | 35 (100.0%) | 8 (80.0%) |  |
| Yes | 0 (0.0%) | 2 (20.0%) |  |
| *Veillonella parvula* |  |  | 0.209 |
| No | 33 (94.3%) | 8 (80.0%) |  |
| Yes | 2 (5.7%) | 2 (20.0%) |  |
| *Streptococcus dysgalactiae* |  |  | 0.065 |
| No | 33 (94.3%) | 7 (70.0%) |  |
| Yes | 2 (5.7%) | 3 (30.0%) |  |
| *Ureaplasma urealyticum* |  |  | 0.539 |
| No | 33 (94.3%) | 9 (90.0%) |  |
| Yes | 2 (5.7%) | 1 (10.0%) |  |
| *Ureaplasma parvum* |  |  | 0.705 |
| No | 25 (71.4%) | 8 (80.0%) |  |
| Yes | 10 (28.6%) | 2 (20.0%) |  |
| *Fusobacterium nucleatum* |  |  | 0.399 |
| No | 34 (97.1%) | 9 (90.0%) |  |
| Yes | 1 (2.9%) | 1 (10.0%) |  |
| *Mycoplasma hominis* |  |  | 1.000 |
| No | 33 (94.3%) | 10 (100.0%) |  |
| Yes | 2 (5.7%) | 0 (0.0%) |  |
| *Sneathia amnii* |  |  | 0.399 |
| No | 34 (97.1%) | 9 (90.0%) |  |
| Yes | 1 (2.9%) | 1 (10.0%) |  |
| *Prevotella enoeca* |  |  | 0.399 |
| No | 34 (97.1%) | 9 (90.0%) |  |
| Yes | 1 (2.9%) | 1 (10.0%) |  |
| *Prevotella fusca* |  |  | 0.399 |
| No | 34 (97.1%) | 9 (90.0%) |  |
| Yes | 1 (2.9%) | 1 (10.0%) |  |
| *Prevotella scopos* |  |  | 0.399 |
| No | 34 (97.1%) | 9 (90.0%) |  |
| Yes | 1 (2.9%) | 1 (10.0%) |  |
| *Prevotella jejuni* |  |  | 0.399 |
| No | 34 (97.1%) | 9 (90.0%) |  |
| Yes | 1 (2.9%) | 1 (10.0%) |  |
| *Megasphaera elsdenii* |  |  | 0.539 |
| No | 33 (94.3%) | 9 (90.0%) |  |
| Yes | 2 (5.7%) | 1 (10.0%) |  |
| *Megasphaera stantonii* |  |  | 0.539 |
| No | 33 (94.3%) | 9 (90.0%) |  |
| Yes | 2 (5.7%) | 1 (10.0%) |  |
| *Lactobacillus vaginalis* |  |  | 0.687 |
| No | 27 (77.1%) | 7 (70.0%) |  |
| Yes | 8 (22.9%) | 3 (30.0%) |  |
| *Lactobacillus crispatus* |  |  | 0.687 |
| No | 8 (22.9%) | 3 (30.0%) |  |
| Yes | 27 (77.1%) | 7 (70.0%) |  |
| *Lactobacillus gasseri* |  |  | 0.087 |
| No | 25 (71.4%) | 10 (100.0%) |  |
| Yes | 10 (28.6%) | 0 (0.0%) |  |
| *Lactobacillus jensenii* |  |  | 0.731 |
| No | 15 (42.9%) | 5 (50.0%) |  |
| Yes | 20 (57.1%) | 5 (50.0%) |  |
| *Lactobacillus iners* |  |  | 1.000 |
| No | 12 (34.3%) | 3 (30.0%) |  |
| Yes | 23 (65.7%) | 7 (70.0%) |  |
| Immune checkpoint protein |  |  |  |
| CD28 | 90.7 (34.6-262.1) | 345.3 (111.9-440.5) | 0.215 |
| TIM-3 | 8.9 (4.1-15.0) | 28.2 (5.2-131.7) | 0.196 |
| HVEM | 429.2 (234.1-632.2) | 548.9 (275.6-750.8) | 0.495 |
| CD40 | 128.4 (60.8-239.9) | 97.1 (61.7-263.4) | 0.978 |
| LAG-3 | 45.7 (33.3-58.2) | 48.8 (23.2-58.2) | 0.965 |
| TLR2 | 132.2 (56.7-269.2) | 200.6 (131.3-235.5) | 0.605 |
| PD-1 | 169.1 (102.7-192.8) | 150.7 (127.4-187.8) | 0.807 |
| CD80/B7-1 | 6.4 (1.3- 9.5) | 18.5 (15.6-25.0) | 0.015 |
| CD86/B7-2 | 5.5 (5.5- 6.8) | 7.4 (6.2- 9.2) | 0.206 |
| PD-L1 | 0.3 (0.2- 1.2) | 0.2 (0.1- 0.6) | 0.424 |
| PD-L2 | 3.3 (0.7-12.3) | 19.0 (18.1-82.8) | 0.046 |
| ICOS | 10.9 (9.4-14.0) | 10.9 (10.9-12.5) | 0.918 |
| Inflammatory cytokine |  |  |  |
| CCL2 | 19.5 (8.5-53.8) | 28.8 (17.3-391.8) | 0.238 |
| CCL3 | 10.7 (9.4-14.3) | 30.7 (7.1-169.8) | 0.266 |
| CCL4 | 102.8 (73.5-145.4) | 113.8 (75.9-289.3) | 0.465 |
| CXCL10 | 2.2 (1.2- 3.0) | 2.3 (0.6-10.7) | 1.000 |
| GM-CSF | 6.6 (5.2-11.0) | 7.5 (4.3-37.4) | 0.739 |
| IFN-α | 2.7 (1.9- 4.5) | 3.0 (2.3- 7.2) | 0.503 |
| IFN-γ | 42.2 (38.9-47.3) | 44.9 (33.8-76.1) | 0.739 |
| IL-1α | 55.8 (34.0-73.6) | 64.9 (22.0-225.1) | 0.724 |
| IL-1β | 128.5 (59.0-418.1) | 297.2 (64.9-751.9) | 0.461 |
| IL-6 | 40.5 (21.3-53.5) | 231.4 (43.3-1841.2) | 0.075 |
| IL-8 | 294.6 (215.1-512.4) | 432.9 (227.3-1888.5) | 0.317 |
| IL-10 | 26.2 (8.7-47.5) | 20.0 (13.9-80.4) | 0.713 |
| IL-12 | 11.7 (8.8-24.8) | 13.9 (7.8-49.7) | 0.718 |
| IL-13 | 22.3 (10.5-36.8) | 26.8 (14.2-43.3) | 0.486 |
| TNF-α | 4.5 (1.9- 8.8) | 5.5 (1.3-61.5) | 0.793 |

**Supplementary Table S14.** Endocervical Spearman's rank correlation Rho values between microbiome and immune-checkpoint proteins

| Variable | Total microbiome  read | *Gardnerella  vaginalis* | *Moraxella  osloensis* | *Lactobacillus  crispatus* | *Lactobacillus  gasseri* | *Lactobacillus  jensenii* | *Lactobacillus  iners* | CD28 | TIM-3 | HVEM | CD40 | LAG-3 | TLR2 | PD-1 | CTLA-4 | CD80/B7-1 | CD86/B7-2 | PD-L1 | PD-L2 | ICOS |
| --- | --- | --- | --- | --- | --- | --- | --- | --- | --- | --- | --- | --- | --- | --- | --- | --- | --- | --- | --- | --- |
| *Total microbiome read* | 1 |  |  |  |  |  |  |  |  |  |  |  |  |  |  |  |  |  |  |  |
| *Gardnerella vaginalis* | -0.61 | 1 |  |  |  |  |  |  |  |  |  |  |  |  |  |  |  |  |  |  |
| *Moraxella osloensis* | 0.04 | -0.25 | 1 |  |  |  |  |  |  |  |  |  |  |  |  |  |  |  |  |  |
| *Lactobacillus crispatus* | 0.58 | -0.51 | 0.02 | 1 |  |  |  |  |  |  |  |  |  |  |  |  |  |  |  |  |
| *Lactobacillus gasseri* | -0.09 | -0.25 | -0.39 | 0.58 | 1 |  |  |  |  |  |  |  |  |  |  |  |  |  |  |  |
| *Lactobacillus jensenii* | 0.33 | -0.34 | -0.52 | -0.04 | -0.02 | 1 |  |  |  |  |  |  |  |  |  |  |  |  |  |  |
| *Lactobacillus iners* | -0.47 | 0.23 | 0.15 | -0.84* | -0.52 | -0.15 | 1 |  |  |  |  |  |  |  |  |  |  |  |  |  |
| CD28 | -0.71 | 0.61 | -0.4 | -0.43 | 0.36 | -0.26 | 0.2 | 1 |  |  |  |  |  |  |  |  |  |  |  |  |
| TIM-3 | -0.43 | 0.41 | -0.80* | -0.29 | 0.18 | 0.33 | 0.26 | 0.36 | 1 |  |  |  |  |  |  |  |  |  |  |  |
| HVEM | -0.22 | -0.21 | -0.54 | 0.2 | 0.79* | 0.03 | 0.04 | 0.4 | 0.52 | 1 |  |  |  |  |  |  |  |  |  |  |
| CD40 | -0.36 | 0 | -0.67 | 0.16 | 0.80* | 0.08 | -0.04 | 0.5 | 0.68 | 0.95*** | 1 |  |  |  |  |  |  |  |  |  |
| LAG-3 | -0.25 | 0.61 | -0.53 | -0.74 | -0.49 | 0.26 | 0.57 | 0.32 | 0.64 | -0.05 | 0.04 | 1 |  |  |  |  |  |  |  |  |
| TLR2 | -0.32 | 0.61 | -0.67 | -0.07 | 0.45 | -0.18 | -0.16 | 0.82* | 0.43 | 0.41 | 0.54 | 0.36 | 1 |  |  |  |  |  |  |  |
| PD-1 | -0.29 | 0.61 | -0.58 | -0.36 | -0.27 | 0.45 | 0.04 | 0.14 | 0.71 | -0.18 | 0.07 | 0.68 | 0.25 | 1 |  |  |  |  |  |  |
| CTLA-4 | -0.33 | 0.62 | -0.75 | -0.58 | -0.2 | 0.48 | 0.24 | 0.44 | 0.76 | 0.03 | 0.22 | 0.87* | 0.49 | 0.87* | 1 |  |  |  |  |  |
| CD80/B7-1 | -0.14 | 0.61 | -0.80* | -0.16 | 0.09 | 0.04 | 0.08 | 0.43 | 0.75 | 0.34 | 0.46 | 0.71 | 0.75 | 0.57 | 0.71 | 1 |  |  |  |  |
| CD86/B7-2 | -0.39 | 0.61 | -0.53 | -0.81* | -0.45 | 0.35 | 0.57 | 0.39 | 0.68 | -0.05 | 0.07 | 0.96*** | 0.32 | 0.75 | 0.93*** | 0.61 | 1 |  |  |  |
| PD-L1 | -0.39 | 0.61 | -0.53 | -0.81* | -0.45 | 0.35 | 0.57 | 0.39 | 0.68 | -0.05 | 0.07 | 0.96*** | 0.32 | 0.75 | 0.93*** | 0.61 | 1.00*** | 1 |  |  |
| PD-L2 | -0.11 | 0.41 | -0.80* | -0.25 | -0.04 | 0.32 | 0.26 | 0.18 | 0.89* | 0.36 | 0.46 | 0.79 | 0.43 | 0.68 | 0.76 | 0.89* | 0.71 | 0.71 | 1 |  |
| ICOS | -0.64 | 0.61 | -0.27 | -0.67 | 0.04 | -0.18 | 0.37 | 0.93* | 0.21 | 0.14 | 0.21 | 0.46 | 0.68 | 0.14 | 0.49 | 0.32 | 0.54 | 0.54 | 0.11 | 1 |

*^*^ P < 0.05; ^**^ P <0.01; ^***^ P < 0.001.*

**Supplementary Table S15.** Endocervical Spearman's rank correlation Rho values between immune-checkpoint proteins and inflammatory cytokines

| Variable | CD28 | TIM-3 | HVEM | CD40 | LAG-3 | TLR2 | PD-1 | CTLA-4 | CD80/B7-1 | CD86/B7-2 | PD-L1 | PD-L2 | ICOS | CCL2 | CCL4 | GM-CSF | IFN-α | IFN-γ | IL-1α | IL-1β | IL-6 | IL-8 | IL-10 | IL-17A | TNF-α |
| --- | --- | --- | --- | --- | --- | --- | --- | --- | --- | --- | --- | --- | --- | --- | --- | --- | --- | --- | --- | --- | --- | --- | --- | --- | --- |
| CD28 | 1 |  |  |  |  |  |  |  |  |  |  |  |  |  |  |  |  |  |  |  |  |  |  |  |  |
| TIM-3 | -0.03 | 1 |  |  |  |  |  |  |  |  |  |  |  |  |  |  |  |  |  |  |  |  |  |  |  |
| HVEM | 0.03 | 0.23 | 1 |  |  |  |  |  |  |  |  |  |  |  |  |  |  |  |  |  |  |  |  |  |  |
| CD40 | 0.2 | 0.49 | 0.93* | 1 |  |  |  |  |  |  |  |  |  |  |  |  |  |  |  |  |  |  |  |  |  |
| LAG-3 | -0.09 | 0.43 | -0.7 | -0.54 | 1 |  |  |  |  |  |  |  |  |  |  |  |  |  |  |  |  |  |  |  |  |
| TLR2 | 0.71 | 0.09 | 0.06 | 0.26 | -0.03 | 1 |  |  |  |  |  |  |  |  |  |  |  |  |  |  |  |  |  |  |  |
| PD-1 | 0.03 | 0.77 | -0.41 | -0.09 | 0.77 | 0.2 | 1 |  |  |  |  |  |  |  |  |  |  |  |  |  |  |  |  |  |  |
| CTLA-4 | 0.09 | 0.62 | -0.58 | -0.26 | 0.79 | 0.18 | 0.97*** | 1 |  |  |  |  |  |  |  |  |  |  |  |  |  |  |  |  |  |
| CD80/B7-1 | 0.09 | 0.6 | -0.06 | 0.14 | 0.54 | 0.6 | 0.66 | 0.53 | 1 |  |  |  |  |  |  |  |  |  |  |  |  |  |  |  |  |
| CD86/B7-2 | 0.03 | 0.49 | -0.7 | -0.49 | 0.94* | -0.09 | 0.83 | 0.88* | 0.37 | 1 |  |  |  |  |  |  |  |  |  |  |  |  |  |  |  |
| PD-L1 | 0.03 | 0.49 | -0.7 | -0.49 | 0.94* | -0.09 | 0.83 | 0.88* | 0.37 | 1.00*** | 1 |  |  |  |  |  |  |  |  |  |  |  |  |  |  |
| PD-L2 | -0.31 | 0.83 | -0.03 | 0.14 | 0.66 | 0.09 | 0.77 | 0.62 | 0.83 | 0.54 | 0.54 | 1 |  |  |  |  |  |  |  |  |  |  |  |  |  |
| ICOS | 0.89* | -0.26 | -0.38 | -0.26 | 0.14 | 0.49 | 0.03 | 0.18 | -0.09 | 0.26 | 0.26 | -0.43 | 1 |  |  |  |  |  |  |  |  |  |  |  |  |
| CCL2 | -0.14 | 0.37 | 0.12 | 0.03 | 0.43 | -0.43 | 0.09 | 0 | 0.09 | 0.37 | 0.37 | 0.37 | -0.09 | 1 |  |  |  |  |  |  |  |  |  |  |  |
| CCL4 | 0.09 | 0.77 | 0.41 | 0.54 | 0.14 | -0.26 | 0.37 | 0.26 | 0.03 | 0.31 | 0.31 | 0.37 | -0.09 | 0.6 | 1 |  |  |  |  |  |  |  |  |  |  |
| GM-CSF | -0.2 | 0.43 | 0.67 | 0.6 | -0.26 | -0.54 | -0.14 | -0.26 | -0.31 | -0.14 | -0.14 | 0.09 | -0.37 | 0.6 | 0.83 | 1 |  |  |  |  |  |  |  |  |  |
| IFN-α | -0.58 | 0.33 | 0.52 | 0.46 | -0.33 | -0.7 | -0.09 | -0.19 | -0.39 | -0.21 | -0.21 | 0.09 | -0.7 | 0.21 | 0.58 | 0.82 | 1 |  |  |  |  |  |  |  |  |
| IFN-γ | -0.26 | 0.09 | 0.43 | 0.37 | -0.49 | -0.66 | -0.26 | -0.26 | -0.71 | -0.26 | -0.26 | -0.31 | -0.31 | 0.09 | 0.54 | 0.77 | 0.88* | 1 |  |  |  |  |  |  |  |
| IL-1α | -0.2 | 0.14 | 0.7 | 0.6 | -0.6 | -0.54 | -0.37 | -0.44 | -0.6 | -0.43 | -0.43 | -0.26 | -0.37 | 0.2 | 0.6 | 0.89* | 0.88* | 0.94* | 1 |  |  |  |  |  |  |
| IL-1β | -0.2 | 0.14 | 0.7 | 0.6 | -0.6 | -0.54 | -0.37 | -0.44 | -0.6 | -0.43 | -0.43 | -0.26 | -0.37 | 0.2 | 0.6 | 0.89* | 0.88* | 0.94* | 1.00*** | 1 |  |  |  |  |  |
| IL-6 | -0.14 | 0.77 | 0.75 | 0.83 | -0.14 | -0.14 | 0.2 | 0 | 0.2 | -0.09 | -0.09 | 0.49 | -0.49 | 0.43 | 0.83 | 0.83 | 0.7 | 0.49 | 0.66 | 0.66 | 1 |  |  |  |  |
| IL-8 | -0.6 | 0.26 | 0.23 | 0.2 | -0.2 | -0.77 | 0.03 | 0 | -0.49 | -0.03 | -0.03 | 0.03 | -0.6 | 0.09 | 0.49 | 0.66 | 0.94* | 0.89* | 0.77 | 0.77 | 0.49 | 1 |  |  |  |
| IL-10 | -0.26 | 0.09 | 0.43 | 0.37 | -0.49 | -0.66 | -0.26 | -0.26 | -0.71 | -0.26 | -0.26 | -0.31 | -0.31 | 0.09 | 0.54 | 0.77 | 0.88* | 1.00*** | 0.94* | 0.94* | 0.49 | 0.89* | 1 |  |  |
| IL-17A | 0.03 | 0.31 | 0.75 | 0.71 | -0.49 | -0.37 | -0.26 | -0.35 | -0.43 | -0.31 | -0.31 | -0.14 | -0.2 | 0.37 | 0.77 | 0.94* | 0.76 | 0.83 | 0.94* | 0.94* | 0.77 | 0.6 | 0.83 | 1 |  |
| TNF-α | 0.09 | 0.77 | 0.41 | 0.54 | 0.14 | -0.26 | 0.37 | 0.26 | 0.03 | 0.31 | 0.31 | 0.37 | -0.09 | 0.6 | 1.00*** | 0.83 | 0.58 | 0.54 | 0.6 | 0.6 | 0.83 | 0.49 | 0.54 | 0.77 | 1 |

*^*^ P < 0.05; ^**^ P <0.01; ^***^ P < 0.001.*

**Supplementary Table S16.** Endocervical Spearman's rank correlation Rho values between immune-checkpoint proteins and inflammatory cytokines

| Variable | *Lactobacillus* | *Gardnerella  vaginalis* | *Moraxella  osloensis* | *Streptococcus  dysgalactiae* | *Ureaplasma  parvum* | *Fusobacterium  nucleatum* | *Sneathia  amnii* | *Prevotella* | *Megasphaera* | *Lactobacillus  vaginalis* | *Lactobacillus  crispatus* | *Lactobacillus  gasseri* | *Lactobacillus  jensenii* | *Lactobacillus  iners* | CD28 | TIM-3 | HVEM | CD40 | LAG-3 | TLR2 | PD-1 | CTLA-4 | CD80/B7-1 | CD86/B7-2 | PD-L1 | PD-L2 | ICOS |
| --- | --- | --- | --- | --- | --- | --- | --- | --- | --- | --- | --- | --- | --- | --- | --- | --- | --- | --- | --- | --- | --- | --- | --- | --- | --- | --- | --- |
| *Lactobacillus* | 1 |  |  |  |  |  |  |  |  |  |  |  |  |  |  |  |  |  |  |  |  |  |  |  |  |  |  |
| *Gardnerella  vaginalis* | -0.85* | 1 |  |  |  |  |  |  |  |  |  |  |  |  |  |  |  |  |  |  |  |  |  |  |  |  |  |
| *Moraxella  osloensis* | 0.39 | -0.11 | 1 |  |  |  |  |  |  |  |  |  |  |  |  |  |  |  |  |  |  |  |  |  |  |  |  |
| *Streptococcus  dysgalactiae* | -0.65 | 0.77 | -0.42 | 1 |  |  |  |  |  |  |  |  |  |  |  |  |  |  |  |  |  |  |  |  |  |  |  |
| *Ureaplasma  parvum* | -0.65 | 0.77 | -0.42 | 1.00*** | 1 |  |  |  |  |  |  |  |  |  |  |  |  |  |  |  |  |  |  |  |  |  |  |
| *Fusobacterium  nucleatum* | -0.65 | 0.77 | -0.42 | 1.00*** | 1.00*** | 1 |  |  |  |  |  |  |  |  |  |  |  |  |  |  |  |  |  |  |  |  |  |
| *Sneathia  amnii* | -0.65 | 0.77 | -0.42 | 1.00*** | 1.00*** | 1.00*** | 1 |  |  |  |  |  |  |  |  |  |  |  |  |  |  |  |  |  |  |  |  |
| *Prevotella* | -0.65 | 0.77 | -0.42 | 1.00*** | 1.00*** | 1.00*** | 1.00*** | 1 |  |  |  |  |  |  |  |  |  |  |  |  |  |  |  |  |  |  |  |
| *Megasphaera* | -0.65 | 0.77 | -0.42 | 1.00*** | 1.00*** | 1.00*** | 1.00*** | 1.00*** | 1 |  |  |  |  |  |  |  |  |  |  |  |  |  |  |  |  |  |  |
| *Lactobacillus  vaginalis* | 0.07 | 0.22 | 0.47 | -0.31 | -0.31 | -0.31 | -0.31 | -0.31 | -0.31 | 1 |  |  |  |  |  |  |  |  |  |  |  |  |  |  |  |  |  |
| *Lactobacillus  crispatus* | 0.83 | -0.85* | 0.39 | -0.65 | -0.65 | -0.65 | -0.65 | -0.65 | -0.65 | -0.27 | 1 |  |  |  |  |  |  |  |  |  |  |  |  |  |  |  |  |
| *Lactobacillus  gasseri* | -0.13 | -0.31 | -0.42 | -0.2 | -0.2 | -0.2 | -0.2 | -0.2 | -0.2 | -0.31 | -0.13 | 1 |  |  |  |  |  |  |  |  |  |  |  |  |  |  |  |
| *Lactobacillus  jensenii* | 0.68 | -0.48 | -0.14 | -0.31 | -0.31 | -0.31 | -0.31 | -0.31 | -0.31 | 0.22 | 0.37 | -0.31 | 1 |  |  |  |  |  |  |  |  |  |  |  |  |  |  |
| *Lactobacillus  iners* | -0.7 | 0.72 | -0.48 | 0.7 | 0.7 | 0.7 | 0.7 | 0.7 | 0.7 | -0.14 | -0.52 | -0.42 | -0.11 | 1 |  |  |  |  |  |  |  |  |  |  |  |  |  |
| CD28 | -0.14 | -0.1 | -0.39 | -0.39 | -0.39 | -0.39 | -0.39 | -0.39 | -0.39 | 0.27 | -0.09 | 0.13 | 0.3 | 0.27 | 1 |  |  |  |  |  |  |  |  |  |  |  |  |
| TIM-3 | 0.26 | -0.1 | -0.03 | -0.39 | -0.39 | -0.39 | -0.39 | -0.39 | -0.39 | 0.78 | -0.14 | -0.13 | 0.68 | -0.09 | 0.6 | 1 |  |  |  |  |  |  |  |  |  |  |  |
| HVEM | 0.37 | 0.1 | 0.15 | 0.39 | 0.39 | 0.39 | 0.39 | 0.39 | 0.39 | 0.07 | 0.09 | -0.65 | 0.51 | 0.09 | -0.54 | 0.09 | 1 |  |  |  |  |  |  |  |  |  |  |
| CD40 | 0.03 | 0.34 | -0.21 | 0.65 | 0.65 | 0.65 | 0.65 | 0.65 | 0.65 | -0.1 | -0.14 | -0.65 | 0.44 | 0.52 | -0.31 | 0.03 | 0.89* | 1 |  |  |  |  |  |  |  |  |  |
| LAG-3 | -0.23 | 0.15 | -0.34 | 0.27 | 0.27 | 0.27 | 0.27 | 0.27 | 0.27 | 0.05 | -0.58 | 0.66 | -0.19 | -0.28 | -0.23 | 0.09 | -0.06 | -0.12 | 1 |  |  |  |  |  |  |  |  |
| TLR2 | 0.2 | 0.03 | -0.39 | 0.13 | 0.13 | 0.13 | 0.13 | 0.13 | 0.13 | 0.3 | -0.14 | -0.39 | 0.85* | 0.33 | 0.37 | 0.71 | 0.54 | 0.66 | -0.03 | 1 |  |  |  |  |  |  |  |
| PD-1 | -0.09 | 0.07 | -0.09 | -0.39 | -0.39 | -0.39 | -0.39 | -0.39 | -0.39 | 0.68 | -0.2 | -0.13 | 0.37 | 0.21 | 0.89* | 0.83 | -0.31 | -0.2 | -0.2 | 0.49 | 1 |  |  |  |  |  |  |
| CTLA-4 | -0.52 | 0.11 | -0.87* | 0.42 | 0.42 | 0.42 | 0.42 | 0.42 | 0.42 | -0.65 | -0.39 | 0.7 | -0.29 | 0.29 | 0.15 | -0.33 | -0.39 | -0.09 | 0.49 | -0.09 | -0.21 | 1 |  |  |  |  |  |
| CD80/B7-1 | -0.26 | 0.07 | -0.21 | -0.39 | -0.39 | -0.39 | -0.39 | -0.39 | -0.39 | 0.51 | -0.26 | 0.13 | 0.14 | 0.21 | 0.94* | 0.66 | -0.6 | -0.43 | -0.12 | 0.26 | 0.94* | 0.03 | 1 |  |  |  |  |
| CD86/B7-2 | 0 | -0.05 | -0.25 | 0.13 | 0.13 | 0.13 | 0.13 | 0.13 | 0.13 | 0.02 | -0.38 | 0.66 | -0.07 | -0.46 | -0.32 | 0.09 | 0.03 | -0.12 | 0.97*** | -0.03 | -0.29 | 0.4 | -0.23 | 1 |  |  |  |
| PD-L1 | 0.2 | -0.57 | -0.52 | -0.53 | -0.53 | -0.53 | -0.53 | -0.53 | -0.53 | -0.03 | 0.12 | 0.66 | 0.38 | -0.28 | 0.7 | 0.46 | -0.52 | -0.46 | 0.25 | 0.23 | 0.46 | 0.43 | 0.58 | 0.28 | 1 |  |  |
| PD-L2 | 0.2 | -0.1 | -0.03 | -0.39 | -0.39 | -0.39 | -0.39 | -0.39 | -0.39 | 0.78 | -0.26 | 0.13 | 0.51 | -0.27 | 0.49 | 0.94* | -0.03 | -0.14 | 0.38 | 0.54 | 0.71 | -0.21 | 0.6 | 0.38 | 0.52 | 1 |  |
| ICOS | 0.03 | -0.14 | -0.1 | 0.14 | 0.14 | 0.14 | 0.14 | 0.14 | 0.14 | -0.29 | -0.15 | 0.7 | -0.32 | -0.55 | -0.58 | -0.33 | -0.03 | -0.21 | 0.83* | -0.39 | -0.64 | 0.42 | -0.52 | 0.89* | 0.09 | -0.03 | 1 |

*^*^ P < 0.05; ^**^ P <0.01; ^***^ P < 0.001.*

**Supplementary Table S17.** Exocervical Spearman's rank correlation Rho values between immune-checkpoint proteins and inflammatory cytokines

| Variable | CD28 | TIM-3 | HVEM | CD40 | LAG-3 | TLR2 | PD-1 | CTLA-4 | CD80/B7-1 | CD86/B7-2 | PD-L1 | PD-L2 | ICOS | CCL2 | CCL4 | GM-CSF | IFN-α | IFN-γ | IL-1α | IL-1β | IL-6 | IL-8 | IL-10 | IL-17A | TNF-α |
| --- | --- | --- | --- | --- | --- | --- | --- | --- | --- | --- | --- | --- | --- | --- | --- | --- | --- | --- | --- | --- | --- | --- | --- | --- | --- |
| CD28 | 1 |  |  |  |  |  |  |  |  |  |  |  |  |  |  |  |  |  |  |  |  |  |  |  |  |
| TIM-3 | 0.3 | 1 |  |  |  |  |  |  |  |  |  |  |  |  |  |  |  |  |  |  |  |  |  |  |  |
| HVEM | -0.5 | 0.3 | 1 |  |  |  |  |  |  |  |  |  |  |  |  |  |  |  |  |  |  |  |  |  |  |
| CD40 | -0.6 | -0.1 | 0.9 | 1 |  |  |  |  |  |  |  |  |  |  |  |  |  |  |  |  |  |  |  |  |  |
| LAG-3 | -0.67 | -0.31 | -0.15 | -0.15 | 1 |  |  |  |  |  |  |  |  |  |  |  |  |  |  |  |  |  |  |  |  |
| TLR2 | -0.1 | 0.5 | 0.9 | 0.7 | -0.41 | 1 |  |  |  |  |  |  |  |  |  |  |  |  |  |  |  |  |  |  |  |
| PD-1 | 0.8 | 0.7 | -0.2 | -0.4 | -0.72 | 0.1 | 1 |  |  |  |  |  |  |  |  |  |  |  |  |  |  |  |  |  |  |
| CTLA-4 | -0.21 | -0.87 | -0.46 | -0.21 | 0.55 | -0.56 | -0.72 | 1 |  |  |  |  |  |  |  |  |  |  |  |  |  |  |  |  |  |
| CD80/B7-1 | 0.9 | 0.4 | -0.6 | -0.7 | -0.56 | -0.3 | 0.9 | -0.36 | 1 |  |  |  |  |  |  |  |  |  |  |  |  |  |  |  |  |
| CD86/B7-2 | -0.6 | -0.1 | -0.1 | -0.2 | 0.97*** | -0.3 | -0.6 | 0.41 | -0.5 | 1 |  |  |  |  |  |  |  |  |  |  |  |  |  |  |  |
| PD-L1 | 0.5 | 0.1 | -0.5 | -0.7 | 0.21 | -0.2 | 0.1 | 0.36 | 0.3 | 0.3 | 1 |  |  |  |  |  |  |  |  |  |  |  |  |  |  |
| PD-L2 | 0.1 | 0.9 | 0.1 | -0.3 | 0.1 | 0.2 | 0.5 | -0.67 | 0.3 | 0.3 | 0.2 | 1 |  |  |  |  |  |  |  |  |  |  |  |  |  |
| ICOS | -0.67 | -0.31 | -0.15 | -0.15 | 1.00*** | -0.41 | -0.72 | 0.55 | -0.56 | 0.97*** | 0.21 | 0.1 | 1 |  |  |  |  |  |  |  |  |  |  |  |  |
| CCL2 | -0.2 | 0.3 | 0.3 | 0 | 0.46 | 0.4 | -0.3 | 0.1 | -0.4 | 0.6 | 0.6 | 0.4 | 0.46 | 1 |  |  |  |  |  |  |  |  |  |  |  |
| CCL4 | -0.67 | -0.21 | 0.87 | 0.97*** | 0 | 0.67 | -0.56 | -0.03 | -0.82 | -0.05 | -0.56 | -0.36 | 0 | 0.15 | 1 |  |  |  |  |  |  |  |  |  |  |
| GM-CSF | -0.67 | -0.21 | 0.87 | 0.97*** | 0 | 0.67 | -0.56 | -0.03 | -0.82 | -0.05 | -0.56 | -0.36 | 0 | 0.15 | 1.00*** | 1 |  |  |  |  |  |  |  |  |  |
| IFN-α | -0.36 | -0.41 | 0.67 | 0.87 | -0.26 | 0.56 | -0.46 | 0.13 | -0.62 | -0.36 | -0.46 | -0.67 | -0.26 | -0.05 | 0.89* | 0.89* | 1 |  |  |  |  |  |  |  |  |
| IFN-γ | -0.6 | -0.1 | 0.9 | 1.00* | -0.15 | 0.7 | -0.4 | -0.21 | -0.7 | -0.2 | -0.7 | -0.3 | -0.15 | 0 | 0.97*** | 0.97*** | 0.87 | 1 |  |  |  |  |  |  |  |
| IL-1α | -0.6 | -0.1 | 0.9 | 1.00* | -0.15 | 0.7 | -0.4 | -0.21 | -0.7 | -0.2 | -0.7 | -0.3 | -0.15 | 0 | 0.97*** | 0.97*** | 0.87 | 1.00* | 1 |  |  |  |  |  |  |
| IL-1β | -0.6 | -0.1 | 0.9 | 1.00* | -0.15 | 0.7 | -0.4 | -0.21 | -0.7 | -0.2 | -0.7 | -0.3 | -0.15 | 0 | 0.97*** | 0.97*** | 0.87 | 1.00* | 1.00* | 1 |  |  |  |  |  |
| IL-6 | -0.4 | 0.5 | 0.1 | -0.2 | 0.67 | 0 | -0.1 | -0.21 | -0.2 | 0.8 | 0.2 | 0.8 | 0.67 | 0.6 | -0.15 | -0.15 | -0.56 | -0.2 | -0.2 | -0.2 | 1 |  |  |  |  |
| IL-8 | -0.9 | 0.1 | 0.6 | 0.5 | 0.67 | 0.3 | -0.6 | -0.05 | -0.8 | 0.7 | -0.3 | 0.3 | 0.67 | 0.5 | 0.56 | 0.56 | 0.15 | 0.5 | 0.5 | 0.5 | 0.7 | 1 |  |  |  |
| IL-10 | -0.6 | -0.1 | 0.9 | 1.00* | -0.15 | 0.7 | -0.4 | -0.21 | -0.7 | -0.2 | -0.7 | -0.3 | -0.15 | 0 | 0.97*** | 0.97*** | 0.87 | 1.00* | 1.00* | 1.00* | -0.2 | 0.5 | 1 |  |  |
| IL-17A | -0.6 | -0.1 | 0.9 | 1.00* | -0.15 | 0.7 | -0.4 | -0.21 | -0.7 | -0.2 | -0.7 | -0.3 | -0.15 | 0 | 0.97*** | 0.97*** | 0.87 | 1.00* | 1.00* | 1.00* | -0.2 | 0.5 | 1.00* | 1 |  |
| TNF-α | -0.56 | 0.21 | 0.97*** | 0.87 | 0 | 0.87 | -0.36 | -0.29 | -0.72 | 0.05 | -0.36 | 0.05 | 0 | 0.46 | 0.89* | 0.89* | 0.68 | 0.87 | 0.87 | 0.87 | 0.15 | 0.67 | 0.87 | 0.87 | - |

*^*^ P < 0.05; ^**^ P <0.01; ^***^ P < 0.001.*
